# Optimization of predictive performance of intrusion detection system using hybrid ensemble model for secure systems

**DOI:** 10.7717/peerj-cs.1552

**Published:** 2023-09-04

**Authors:** Qaiser Abbas, Sadaf Hina, Hamza Sajjad, Khurram Shabih Zaidi, Rehan Akbar

**Affiliations:** 1 University of Engineering and Technology, Lahore, Pakistan; 2 University of Salford, Salford, UK; 3 University of Engineering and Technology Lahore, Lahore, Pakistan; 4 COMSATS University Islamabad, Lahore, Pakistan; 5 Computer and Information Sciences Department, Universiti Teknologi PETRONAS, Seri Iskandar, Malaysia

**Keywords:** Predictive modelling, Ensemble method, Intrusion detection, Secure systems

## Abstract

Network intrusion is one of the main threats to organizational networks and systems. Its timely detection is a profound challenge for the security of networks and systems. The situation is even more challenging for small and medium enterprises (SMEs) of developing countries where limited resources and investment in deploying foreign security controls and development of indigenous security solutions are big hurdles. A robust, yet cost-effective network intrusion detection system is required to secure traditional and Internet of Things (IoT) networks to confront such escalating security challenges in SMEs. In the present research, a novel hybrid ensemble model using random forest-recursive feature elimination (RF-RFE) method is proposed to increase the predictive performance of intrusion detection system (IDS). Compared to the deep learning paradigm, the proposed machine learning ensemble method could yield the state-of-the-art results with lower computational cost and less training time. The evaluation of the proposed ensemble machine leaning model shows 99%, 98.53% and 99.9% overall accuracy for NSL-KDD, UNSW-NB15 and CSE-CIC-IDS2018 datasets, respectively. The results show that the proposed ensemble method successfully optimizes the performance of intrusion detection systems. The outcome of the research is significant and contributes to the performance efficiency of intrusion detection systems and developing secure systems and applications.

## Introduction

The increase in the active internet users has set off significant risks to the network resources and communication security (Abdulhammed et al., 2018). Any unauthorized activity by an illicit individual, triggering changes to the network resources and systems, stealing sensitive/valuable network resources, or malicious actions to disrupt normal routine of the network is considered as intrusion (Saeed, 2022). A network intrusion detection system has the ability to prevent, detect and respond to such attacks (Belouch, Hadaj & Idhammad, 2018; Bhosale, Nenova & Iliev, 2018). The emerging network technologies, continuous connectivity, diversity in the types and use of networks and continuously evolving and intelligent techniques of intrusions have aggravated the vulnerabilities in the existing systems. In addition, uncontrolled use of personal devices connected with home and office networks during COVID-19, and dependency on emerging digital technologies and trends also opened many vulnerabilities and opportunities for intruders to access organizational networks and systems. Such emerging digital technologies and trends led to many new challenges related to the network and system’s security leaving intrusion detection still as an important open problem (Chen, Fu & Zheng, 2022).

The intrusion detection systems are implemented at two levels as the name depicts such as host-based intrusion detection system (HIDS) and network-based intrusion detection system (NIDS). NIDS are further two categories for anomaly-based and signature-based detections. A signature-based intrusion detection system works with the predefined vulnerabilities and malware signatures which result in limited and inflexible detection whereas anomaly-based systems are dynamic and tend to search the attacks that were not predefined through detection rules. However, this mechanism may result in increased false positive notifications. Yet, it can timely inform the administrator about the possible attack without first recognizing the actual type of attack. Hence, comparatively, it performs well than the signature-based systems (Xu et al., 2021).

The NIDS should perform in real-time and work with maximum accuracy (Zhang et al., 2022). Earlier, intrusion detection systems were signature-based and performed well on the known attacks that were already available in the system’s database. For detection of a new attack, the database needed to be updated on a regular basis. This drawback prevented the systems from working in real-time environments where new attacks are always inevitable. To solve such issues, much research and investigations have been made and still it is required to develop real-time, speedy, yet accurate network anomaly detection systems that can monitor, analyze and detect anomalies in sensitive environments with compact traffic (Otair et al., 2022). However, such systems may get costly if complex computational models are incorporated. To protect large number of networked resources in less privileged enterprises, it is mandatory to design a solution which is not only dynamic but also cost-effective for maximum affordability.

With the increase in the amount of data, machine learning techniques are being overwhelmingly used because of the augmented computational power of various machine learning algorithms to process huge datasets. Machine learning techniques for the development of network intrusion detection systems have been quite effective, however, further research for specific business and organizational settings implemented with distinctive attack vectors is required (Gulla et al., 2020; Injadat et al., 2020; Taher, Jisan & Rahman, 2019). The data mining approach can be used for classification of the network traffic by extracting huge datasets, mining patterns, and performing pattern analysis. Likewise, machine learning models can also be used to classify the attacks in real-time whereby speed and accuracy should further be enhanced to work in challenging environments.

Deep learning technology has widely been used in the development of the NIDS for real-time systems (Abdel-Basset et al., 2021; Hussien, 2020). Its effective feature extraction ability from the raw data has made this a popular practice for the development of automated systems. Recent research has shown that although it works better for network anomaly detection and outperforms the traditional machine learning and rule-based methods, it still has some drawbacks. First, it requires huge, labeled datasets which are mostly not available publicly. Secondly, this technology is computationally expensive and requires costly graphic processing units (GPUs) and other computing resources (Abdel-Basset et al., 2021). Additionally, the deep learning-based system is vastly complex and hard to interpret. In recent years, Internet of Things (IoT) and edge computing has been the focus for the communication between various computing and networking devices (de Souza, Westphall & Machado, 2022). Many traditional computing systems don’t have high-end computing resources, therefore, the deep learning methods might also not be fully utilized in the IoT and edge computing domain due to resources limitations (Nasir et al., 2022). Moreover, small and medium enterprises (SMEs) generally lack the required resources, and skills to combat cyber-attack vectors, yet they need to utilize some methods to stay competitive and secure. Newer methods/technologies, to be adopted by developing SMEs, are valuable only if they are cost-effective and robust in resisting latest unknown network attacks.

Network intrusion detection is very crucial for organizations to safeguard against data theft. Traditional intrusion detection methods work on rule-based systems where manual analysis of network traffic is quite a challenging problem. Although machine learning based methods can help in learning the traffic behavior from historical data, however, working with real time traffic data requires manual feature selection which requires time and domain expertise. Traffic data is usually high dimensional and determining important features is an important step for predictive systems. In addition, simple machine learning algorithms can struggle while working with high dimensional data. Ensemble learning is a technique in which multiple algorithms are used for prediction. Ensemble learning helps in increasing the predictive performance for a given problem. Therefore, an ensemble method which could be integrated with important features selection techniques, is required for developing high performance predictive methods for network intrusion detection.

In this article, a hybrid ensemble method is proposed for the development of an efficient cost-effective network intrusion detection system. Based on the evaluation of the performance of major machine learning and deep learning algorithms on four performance parameters, including accuracy, precision, recall, and F1-score, a cost-effective model is proposed to optimize the predictive performance. One of the major challenges in evaluating performance of machine learning models is the unavailability of large datasets. In previous research works, KDD CUP 99 dataset (University of California at Irvine, 2022) has been prominently used which is outdated and does not depict real attacks in the networks. The present research work uses Network Security Laboratory– Knowledge Discovery and Datamining (NSL-KDD) (Canadian Institute of Cybersecurity, 2022), University of New South Wales-New Brunswick (UNSW-NB15) and Communications Security Establishment-Canadian Institute of Cybersecurity (CSE-CIC-IDS2018) datasets. Data preprocessing steps were applied on the dataset to make it compatible with the algorithm requirements. Additionally, a feature selection method has been implemented to select the best input features for the selected classification algorithms. Conclusively, the ensemble model optimized the prediction of domain specific attacks and contributed to the design of secure networks systems, and applications.

## Literature review and related work

During recent years, artificial intelligence techniques, due to their powerful automated feature extraction methods, have been widely implemented to develop solutions in the broader domain of security. During the last decade, many researchers have used machine learning and artificial intelligence algorithms for detection of network intrusion. Various learning-based methods have been proposed to improve the performance of intrusion detection systems. In this section a thorough review of related work is presented where latest algorithms and techniques have been studied to identify the prospects of improvement in terms of efficiency, accuracy, and cost-effectiveness of intelligent network intrusion detection methods.

Abdulhammed et al. (2018) proposed a machine learning method for wireless network intrusion detection system that included classification and feature selection methods. They first preprocessed the data to feed the model and later applied various algorithms such as AdaBoost (adaptive boosting), random forest (RF), and multilayer perceptron (MLP), where the focus was to improve the feature reduction in input data for classification algorithms. This helped in increased speed and detection accuracy. Four feature sets were selected and applied to the models for training. The results showed that RF performed better using 32 input features. The final model developed in this research work resulted in 99% accuracy, 0.99 precision and 0.966 recall on test dataset. The developed system was later applied on an AWID dataset for real-time intrusion detection. Researchers also compared the performance of machine learning methods to show that the proposed and developed model was performing well on the test dataset. Belouch, Hadaj & Idhammad (2018) conducted a research study to evaluate the performance of four classification methods for intrusion detection in networks. They evaluated support vector machine (SVM), Naive Bayes (NB), RF, and decision tree algorithms for the desired output. Apache Spark tool was used to classify the intrusion traffic, whereas, publicly available dataset, UNSW-NB15, with 42 input features for training the machine learning models was used. The experiments showed that RF outperformed the other models and achieved an accuracy of 97% on test data with 93% sensitivity and a specificity of 97%. Further, in the same year, Bhosale, Nenova & Iliev (2018) presented a new filter-based feature selection algorithm which was based on hybrid approach for feature selection. A subset of features was optimized by the hybridized feature selection approach (HFSA) for building a classification algorithm for multi-class classification task. The model was trained using real-time packets which were captured using the JPCAP package. Researchers used a Naive Bayes algorithm for classification of normal attacks. Before feeding the data to the model, it was preprocessed with two methods. First, the data was converted to numerical values and then applied to a phase of data normalization where features were scaled between 0 and 1. Then feature selection was applied along with the Naive Bayes classifier to classify six types of attacks that were normal, remote to user (R2L), denial of service (DoS), user to root (U2R), brute force attacks, and probe. The proposed HFSA algorithm was applied for classification enhancement. The final model achieved an accuracy of 92% while 95% precision and 90% recall values were recorded.

Later, Kim et al. (2020) developed a distributed denial of service (DDoS) detection and classification system using convolutional neural network (CNNs) and compared the performance with recurrent neural networks (RNNs). The system was evaluated using KDDCup99 and CSE-CIC-IDS2018 to differentiate the malicious DDoS traffic from normal network traffic. On KDDCup99, an average accuracy of 99.9% was achieved while CSE-CIC-IDS2018 yielded 91.997% average accuracy for binary and multi-class classification of DDoS traffic. The RNN accuracy was 99% for binary classification but for multi-class classification RNN could only achieve 93% accuracy, on KDDCup99. CNN also outperformed the RNN on CSE-CIC-IDS2018 dataset with 91.3% accuracy as compared to 65% accuracy, respectively. Here, researchers only discussed DDoS categorization while neglecting other classes of datasets. Moreover, convolution operation is very costly, and it increases complexity of the overall system. Injadat et al. (2020) introduced a multi-stage optimized NID framework using optimized ML algorithms with lesser complexity and increased detection performance. Additionally, they studied the impact of oversampling, training size, and information gain and correlation-based feature selection techniques. The framework consisted of a combination of random search, particle swarm optimization (PSO), genetic algorithm (GA) for feature selection and KNN and RF classifiers. The performance was evaluated on CICIDS2017 and UNSW-NB-15 datasets with more than 99% accuracy on both datasets. The framework required only 74% of training sample size and 50% features for training. The proposed work was a multi-stage framework where each module was dependent on the previous one, which highlighted the need for an end-to-end system with fewer dependencies.

Pokharel, Pokhrel & Sigdel (2020) presented an IDS which was based on hybrid machine learning classification algorithm. Profile improvement methods were applied to improve the detection of abnormal user behavior. The hybrid method was based on Naive Bayes classifier and SVM for abnormal behavior detection. Data preprocessing was also applied on the raw input data. These feature normalization, feature scaling and feature selection methods improved the performance of IDS by achieving an accuracy of 93.1% and a precision of 95.8%. Chkirbene et al. (2020) proposed a dynamic intrusion detection and classification method using feature selection technique. They develop two methods for intrusion detection namely trust-based intrusion detection and classification system (TIDCS) and trust-based intrusion detection and classification system-accelerated (TIDCS-A). They utilized feature selection algorithm to reduce the input features. High rank features are selected to train the system for optimal performance. Both algorithms were trained using NSL-KDD and UNSW datasets and experiments showed that they could detect attacks with greater accuracy and less false alarm rate. The final model achieved an accuracy of 91% using the TICDS method. Xu et al. (2020) presented a new intrusion detection system log-cosh conditional variational autoencoder (LCVAE). It utilized properties of CVAE, and a new loss function log-cosh was introduced which balanced the generation and reconstruction of intrusion data for data classes with fewer samples. For classification and feature extraction, CNN was used, which achieved an accuracy of 85.51% on NSL-KDD dataset. The research considered binary classification while using expensive DL methods.

Gu & Lu (2021) used SVM with Naive Bayes feature embeddings to develop an intrusion detection system. Naive Bayes algorithm was utilized to transform features for data state conversion. SVM model was trained as a classifier. The method was applied to many datasets including UNSW-NB15, NSL-KDD, Kyoto 2006+ and CICIDS2017. Various features were selected from these datasets for training the classifier. The final system was compared with a simple SVM model. This comparison showed that performance of SVM increases by using Naive Bayes embeddings. The best results were obtained using NSL-KDD dataset and the highest accuracy of 99.36% was achieved with a detection rate of 99.25%. Gu & Lu (2021) proposed to enhance the quality of the data for intrusion detection problems. They argued that quality of data can help in the development of robust intrusion detection systems. In their research, they utilized Naive Bayes feature transformation technique to enhance the data quality. Later, they trained SVM classifier on the transformed dataset. The accuracies were 93.75%, 98.92%, 99.35% and 98.58% on UNSW-NB15, CICIDS2017, NSL-KDD and Kyoto 2006+ datasets respectively. However, researchers only considered binary classification where the system could only differentiate between normal and attack traffic with no information about the class of the attack. In addition, they extracted random data to balance the dataset instead of utilizing the complete dataset. Zhao et al. (2021) developed an efficient network intrusion detection using combination of convolutional network and dynamic autoencoders. They also presented a new loss function for NID to train autoencoder and classifier together. The lightweight structural design helped to extract the efficient feature extraction which achieved high accuracy of 93.1% and 98.5% on KDD99 and UNSW-NB15 respectively. Yet gain, researchers only consider binary classification problem and focused on development of lightweight NID method.

Abdel-Basset et al. (2021) proposed a semi-supervised deep learning based intrusion detection system in IoT networks. Researchers introduced a multiscale residual temporal convolutional module (MS-Res) to help network learn the spatiotemporal representations. To improve the importance of influential features, a traffic attention (TA) mechanism was developed. The system was evaluated using CICIDS2017 and CICIDS2018 datasets with more than 99% accuracy in case of binary class classification scenario. Researchers supported the claim that deep learning requires more data and computational cost. Xu et al. (2021) proposed a new five-layer autoencoder model for network anomaly detection. The data bias, due to redundant samples, was removed by preprocessing the data and outliers were removed to reduce the effect of detection bias. It was trained on NSL-KDD and achieved an accuracy of 90.61% and 92.26%. Consideringly, autoencoder is computationally expensive deep learning model and the system could only differentiate binary classification problems.

Recently, in 2022, a research study presented a network behavior anomaly detection method based on deep belief network. The method worked by extracting features using deep belief network (DBN) and dimensions were reduced. A DBN was pre-trained on small training data using unsupervised learning and then it was again trained using supervised learning to extract useful features. Later, a light long short-term memory (LSTM) network was used to classify the network anomalies. The proposed model was evaluated using KDD99 with 94% accuracy and CICIDS2017 with 86.8% accuracy. The performance of the proposed model was not good in classes with fewer records. Moreover, in another limitation, two models were needed to train. Therefore, an end-to-end model is required to reduce false alarm rates and enhanced performance (Chen, Fu & Zheng, 2022). Roy et al. (2022) introduced a cyber-attacks detection model for IoT networks. They preprocessed the data using removal of redundant features, sampling the dataset, and reducing dimensions of the dataset. These steps helped in choosing the most relevant features for intrusion detection. In addition, they used the B-Stacking method which is a combination of boosting and stacking algorithms. Researchers utilized CICIDS2017 and NSL-KDD dataset for evaluation. The proposed system has a high detection rate and low false alarm rate with 98.5% and 99.11% accuracy on NSL-KDD and CICIDS2017 datasets respectively. However, research presented lower results on U2R and R2L and the proposed model was only for IoT networks. In a similar research (de Souza, Westphall & Machado, 2022), the authors presented a two-step ensemble approach for intrusion detection in IoT and fog computing. In the first step they used an extra tree as a binary classifier to analyze the traffic and later an ensemble based on ET, RF and DNN was used to detect intrusion traffic. Researchers performed experiments with Bot-IoT, NSL-KDD, IoTID20 and CICIDS2018 datasets. This approach achieved an average precision of 100% with 100% recall value on bot-IoT dataset. On NSL-KDD dataset they achieved 99.81% accuracy with 99.81% precision. Again, low results on U2R (68.75) and R2L (96.31) were reported on NSL-KDD dataset. Fewer attacks related to IoT networks were presented, with no countermeasures.

Zhang et al. (2022) introduced RANet based on group gating convolutional networks. In the last maxpooling layer, they applied an overlapping method and tested the proposed model on five publicly available datasets. It achieved an accuracy of 83.23%, 69.04%, 99.78%, 97.55%, and 96.73% on NSL-KDD Test (+), NSL-KDD (21), KDDCUP99, Kyoto and CICIDS2017 datasets respectively. However, the proposed system showed low performance on infrequent network attack types and had weak interpretability. Rashid et al. (2022) worked on the development of tree-based stacking algorithm using DT, RT and XGBoost. In addition, the effectiveness of k-best model for features selection was analyzed. Researchers evaluated the system using NSL-KDD and UNSW-NB15 datasets with 20 features based on their score. An average accuracy of 93.7% and 99% was achieved on UNSW-NB15 and NSL-KDD datasets respectively. But the authors only considered binary classification problem due to which the system could not differentiate between various attack classes. Another research work utilized CNN for feature extraction and meta-heuristic LSTM for detection of DDoS attack. For effective feature selection and minimizing the correlation among features, they developed a closest position grey wolf optimization (CP-GWO) algorithm with less complexity and better convergence. To evaluate the performance of model DARPA1998, DARPA LLS DDoS-1.0, CICIDS2017, NSL-KDD and KDD99 datasets were used with 96.52%, 95.94%, 96.52%, 96.37% and 96.37% respectively. The research work focused on binary classification of DDoS *vs* benign network traffic where performance on NSL-KDD is comparatively lower (Dora & Lakshmi, 2022).

Nasir et al. (2022) presented an intelligent framework (DF-IDS) to secure edge IoT using deep learning. The framework presented two stages, where first stage started by selecting features using spider monkey (SM), PCA, information gain (IG) and correlation attribute evaluation (CAE). The selected features were fed to a deep neural network. The framework was tested using NSL-KDD dataset and achieved an accuracy of 99.23% and F1-Score of 99.27%. Again, this research worked on binary classification where the system could only differentiate between normal and attack traffic with no information about the type of attack. Otair et al. (2022) presented a wireless sensor networks intrusion detection system using grey wolf optimizer (GWO) and particle swarm optimization (PSO). Researchers selected important features using GWO and utilized PSO to attain global optimum values for selected features. To classify the network traffic an ensemble algorithm consisting of KNN and SVM was used. NSL-KDD was utilized to evaluate the performance of the proposed system with 98.97% accuracy. This also works on binary classification only. Another research Saeed (2022) proposed a hybrid system for real-time intrusion detection of streaming data. Researchers selected 16 features from input data that contributed to the performance enhancement. For classification, KNN, Naive Bayes and a hybrid classifier were used on NSL-KDD and KDD99 datasets with more than 99% accuracy. This system also considers binary classification problem while neglecting information about the type of attack.

Table 1 summarizes the work reviewed in this study including authors, dataset information, pre-processing techniques, feature selection methos, classification techniques, number of features used, evaluation metrics and limitations.

**Table 1:** Critical analysis of IDS methods in relevant literature.

Authors	Dataset	Data pre-processing	Features selection method	Classifier	Classification	No of features used	Evaluation metrics	Limitation
Roy et al. (2022)	CICIDS2017, NSL-KDD	Dimensions reduction	–	B-Stacking ensemble	Multi-class	28	Accuracy 98.5%	Low performance on U2R and R2L classes.
de Souza, Westphall & Machado (2022)	BoT-IoT, NSL-KDD, IoTID20, CICIDS2018	Standard scaling, SMOTE	Extra tree	Ensemble of ET, RF and DNN	Multi-class	20	Accuracy 99.81%, Precision 99.81%	Low performance on U2R and R2L, Fewer IoT related attacks
Zhang et al. (2022)	NSL-KDD, KDD99, CICIDS2017	MinMax normalization	CNN	CNN based RANet	Multi-class	41 and 122	Accuracy 83.23%	Poor performance on infrequent attack types
Rashid et al. (2022)	NSL-KDD, UNSW-NB15	MinMax normalization	k-best model	Ensemble of RF, XGBoost and DT	Binary	20	Accuracy 99%	No information about attack classes
Dora & Lakshmi (2022)	NSL-KDD, DARPA1998, DDoS-1.0, KDD99	Correlation minimization	CP-GWO (Closest Position)	CNN + LSTM	Binary	5	Accuracy 96.37%, Precision 97.44%, Recall 98.78%	Specifically designed for DDoS detection
Nasir et al. (2022)	NSL-KDD	Data normalization	Spider monkey (SM), PCA, IG	Deep neural network	Binary	14	Accuracy 99.23%, Precision 99.30%, Recall 99.24%, F1-Score 99.27%	No information about types of attacks
Otair et al. (2022)	NSL-KDD	Data normalization	GWO + PSO	Ensemble of KNN + SVM	Binary	20	Accuracy 98.97%, Detection Rate 98.57%	Can only distinguish between attack and benign traffic.
Chen, Fu & Zheng (2022)	KDD99, CICIDS2017	Data normalization	Deep belief network	LSTM	Multi-class	–	Accuracy 94.25%	Low performance on U2R and R2L classes
Saeed (2022)	NSL-KDD, KDD CUP 99	–	Minimum redundancy—Maximum relevance MRMR	KNN + Naïve Bayes	Binary	16	Accuracy 99%, Precision 99.7%, Recall 99.75%	Neglects additional attack information
Injadat et al. (2020)	CICIDS2017, UNSW-NB15	Z-Score normalization, SMOTE	Information Gain, PSO, GA	KNN + RF	Multi-class	31 and 41	Accuracy 99%, Precision 98%, Recall 99%	Complex module-based architecture
Gu & Lu (2021)	UNSW-ND15, CICIDS2017, NSL-KDD, Kyoto 2006+	Naïve Bayes feature embeddings	–	SVM	Binary	–	Accuracy 99.35%, Detection Rate 99.25%	Use a part of data instead of whole dataset, only consider binary classification problem
Abdel-Basset et al. (2021)	CICIDS2017, CICIDS2018	Redundant feature elimination, Data normalization	Traffic Attention	Modified residual network	Multi-class	–	Accuracy 99.6%, Precision 92.31%, Recall 96.29%	Additional computational cost due to DL
Zhao et al. (2021)	KDD99, UNSW-NB15	Data normalization, PCA	CNN	CNN + Dynamic autoencoder	Binary	–	Accuracy 93.1%, Precision 99.8%, Recall 91.6% (on KDD99)	Focus on lightweight model development and classification performance is very low.
Xu et al. (2021)	NSL-KDD and UNSW	Outlier analysis, Data normalization	–	Autoencoder	Binary	122	Accuracy 90.61%, Precision 86.83%, Recall 98.34%, F1-Score 92.26%	Cannot differentiate subclasses of the attack types
Kim et al. (2020)	KDD99, CICIDS2018	–	CNN	Fully connected network	Binary	–	Accuracy 99.9%, Recall 100%, Precision 99.9% (KDD99)	Costly convolution operation + Special system for DDoS detection
Xu et al. (2020)	NSL-KDD	Data balancing using log-cosh function	CNN	Conditional variational autoencoder	Binary	–	Accuracy 85.51%, Precision 97.62%, Recall 68.90%	Expensive DL method + no information about attack classifications

**Note:**

AWID, Aegean Wi-Fi Intrusion Dataset; MLP, Multi-Layer Perceptron; UNSW-NB15, University of New South Wales; SVM, Support Vector Machine; KDD, Knowledge Discovery in Databases; HFSA, Hybrid Feature Selection Algorithm; SDN, Software Defined Networking; KNN, k-Nearest Neighbors; PCA, Principal Components Analysis; CIC, Canadian Institute for Cybersecurity; LSTM, Long Short-Term Memory; CNN, Convolutional Neural Network; SMOTE, Synthetic Minority Oversampling Technique; GWO, Grey Wolf Optimizer; PSO, Particle Swarm Optimization.

The table presents the summarized relevant information. Some of the problems with the recent work are discussed based on classification type, dataset choice, required computational resources, training complexity and low results on minority classes.

In the literature, majority of the researchers have worked on binary classification of intrusion traffic (Gu & Lu, 2021; Zhao et al., 2021; Rashid et al., 2022; Dora & Lakshmi, 2022) while some also worked on multi-class classification using deep learning techniques (Chen, Fu & Zheng, 2022; Zhang et al., 2022; Abdel-Basset et al., 2021; Moizuddin & Jose, 2022). However, deep learning requires computational resources and time to train the models (Gu & Lu, 2021). This claim is also supported by results of the deep learning algorithm experiments performed in this research. In addition to that, large, labeled datasets are also a primary requirement to train deep learning algorithms. Although some of the researchers have used simple ML based approaches but they utilized older dataset such as KDD99 (Saeed, 2022; Zhang et al., 2022; Dora & Lakshmi, 2022). Moreover, in some recent studies (Chen, Fu & Zheng, 2022; de Souza, Westphall & Machado, 2022; Roy et al., 2022), detailed analysis and classification of network intrusion is done using machine learning, still, they achieved low results on minority classes. Besides, various researchers (Injadat et al., 2020; Gupta, Jindal & Bedi, 2022) developed complex module-based approaches that cannot be directly used on low-end devices.

To fill the gap in literature, an end-to-end lightweight and cost-effective machine learning approach for optimized network intrusion detection is developed, by utilizing latest intrusion datasets to evaluate the approach. As compared to the recent studies, the proposed method is simple, computationally efficient, works on multi-class classification and does not require high-end computational resources. The popular deep learning algorithms such as CNN, RNN and LSTM were also trained to compare the performance of the proposed system with the deep learning-based methods. The proposed method gives results that are comparable to the latest deep learning approaches and has some considerable advantages over deep learning-based approaches.

## Methodology and evaluations

In the proposed method, data normalization technique, and a wrapper-based feature elimination method are utilized to select 13 and 15 important features from NSL-KDD and other two datasets, respectively. Only 31%, 33% and 21% features for NSL-KDD, UNSW-NB15 and CSE-CIC-IDS2018 datasets were used, respectively. In the next step, a hybrid stacking ensemble was developed using RF-RFE, MLP, Random Forest and SVM for classification. A majority voting mechanism was utilized to calculate the probabilities of attack classes. The results indicated the superiority of the proposed approach for network intrusion detection.

**Figure 1:**
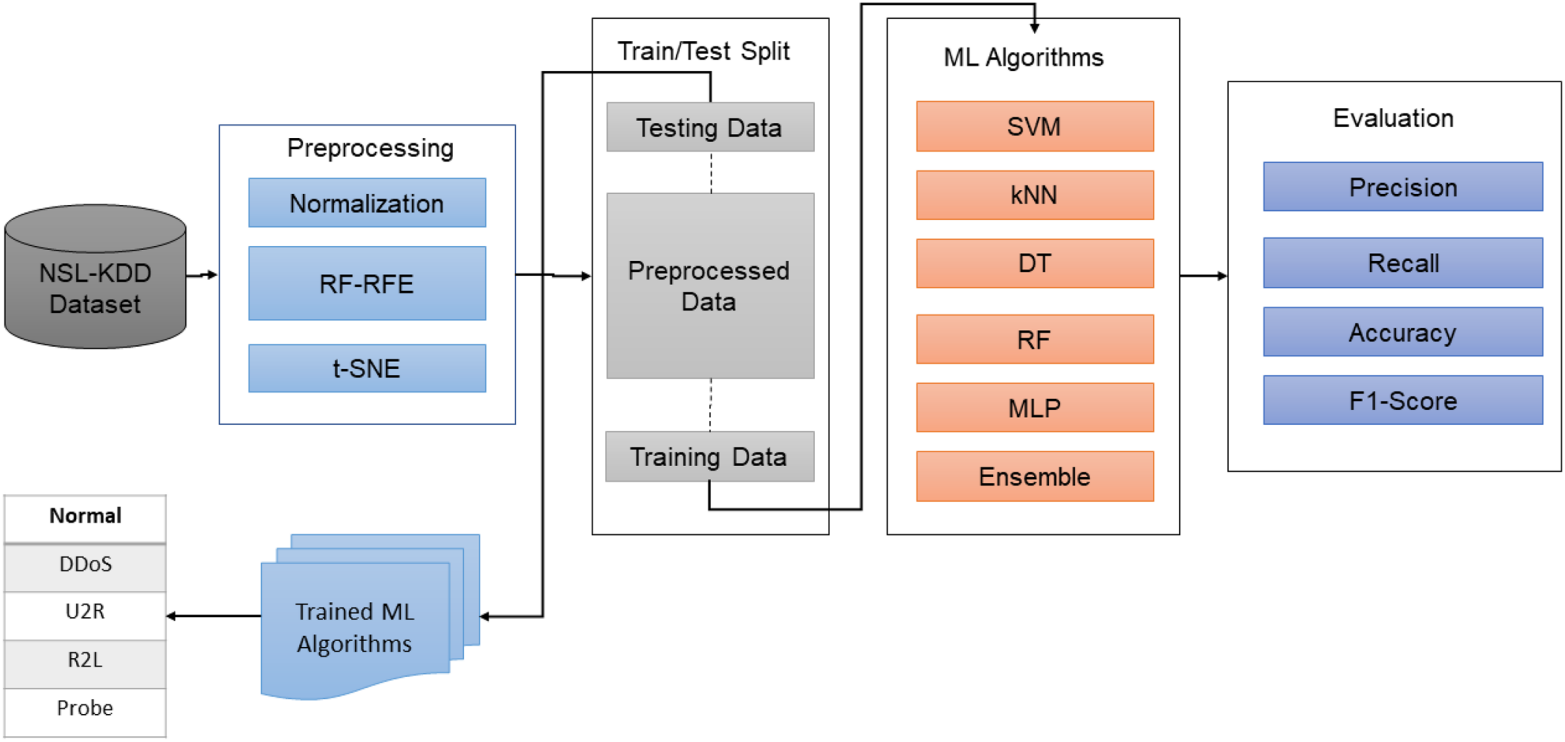
The proposed methodology.

For this study, datasets were downloaded from public repositories. The input data was normalized, and features were reduced using a hybrid method *i.e*., RF-RFE. In the next step, the datasets were again saved in the reduced form, and were split into training and testing parts. Initially, major machine learning algorithms were utilized to learn the attacks behavior and classes. However, due to complex nature and high-dimensional data, single algorithms didn’t work quite well. To overcome this issue, the proposed hybrid ensemble algorithm was trained on preprocessed datasets to learn the attack types. To evaluate the system, widely used classification metrics were utilized, as shown in Fig. 1.

### Dataset description

The characteristics and features of datasets used in the evaluation of the proposed mode have been presented as follows.

### NSL-KDD

In related work, most of the researchers used KDDCup99 for training their final systems. Although this dataset is good to train the classifiers, but it has some issues such as duplicate values and lack of real-world attacks scenarios. To develop a practically applicable robust intrusion detection system, this research work primarily used a newer dataset, NSL-KDD, to validate the performance of the proposed method, as it had real-world scenarios and millions of network records for NID evaluation. This dataset does not have redundant records, so any model trained on this dataset should not be biased towards repeated attack records. The NSL-KDD (Canadian Institute of Cybersecurity, 2022) consists of four sub-datasets that are KDD Test+, KDD Train, KDD Test-21, and _20 Percent. The dataset has records of various network attacks that an intrusion detection system must detect to avoid security problems. There are total of 43 features in this dataset for a single record. Out of 43 features, 41 are related to traffic input and the other two are labels and scores of traffic input. This dataset has a total of four classes for various attacks: probe, user to root (U2R), denial of services (DoS), and remote to local (R2L). A breakdown of number of records distributions is presented in the Table 2.

**Table 2:** Attack types distribution in the NSL-KDD dataset.

Dataset	Number of records					
	Total	Normal	DoS	Probe	U2R	R2L
KDDTrain+20%	25,192	13,449	9,234	2,289	11	209
KDDTrain+	125,973	67,343	45,927	11,656	52	995
KDDTest+	22,544	9,711	7,458	2,421	200	2,654

As mentioned, a DoS attack tries to shut down the system while the other three attacks are related to gaining access to system and sensitive information. This dataset suffers class imbalance problem where more than half of the records are normal. Distribution of U2R and R2L attacks is very low as compared to DoS attack. This property of the dataset resembles the real-life scenarios where DoS is the main type of attack that is a threat to the network and a smaller number of U2R and R2L attacks are seen in real environments. The input features in the dataset can be divided into four main categories that are: intrinsic, content, time-based, and host based.

### UNSW-NB15

This dataset was created by the Australian Center for Cyber Security (Moustafa & Slay, 2015) having more than two million records, 48 features and nine different attack types. These features were extracted using newly developed algorithms such as Bro-IDS and Argus tools. The dataset was generated in the Cyber Range Lab where realistic state-of-the-art normal, and synthetic abnormal networks were established. The features are further categorized into five major groups which are flow features, basic features, content features, and time features along with additional generated features. The dataset contains nine types of different attacks: Fuzzers, Analysis, Backdoors, DoS, Exploits, Generic, Reconnaissance, Shellcode and Worm.

Table 3 presents the overall statistics of the UNSW-NB15 dataset.

**Table 3:** Attack distribution in the UNSW-NB15 dataset.

Attack category	Number of events	Attack category	Number of events	Attack category	Number of events
Fuzzers	24,246	Analysis	2,677	Exploits	44,525
Reconnaissance	13,987	Backdoors	2,329	Generic	215,481
Shellcode	1,511	DoS	16,353	Worms	174

### CSE-CIC-IDS2018

The dataset was created by Communication Security Establishment (CSE) and Canadian Institute of Cybersecurity (CIC) in 2018. This work was aimed to develop user-profile based diversified instructional data for intrusion detection on networks. The data resembles the actual user behavior on the network. The dataset has seven different attack classes: DoS, DDoS, web attacks, botnet, heartbleed, brute-force, and infiltration. Due to the huge amount of dataset, only a part of data was used. CSV files, only from Friday-02-03-2018 and Friday-16-02-2018, were selected for the evaluation purposes. As the data is highly imbalanced, only those parts of the data were selected that could simulate real-world attack scenarios. The selected data has four classes: normal traffic, botnet, DoS-lowHTTP and DoS-Hulk, which are merged to represent a single class *i.e*., attack. After removing null values and duplicate records, 1,074,342 records were retained, out of which 290,089 records were malicious. Table 4 provides overall details of the dataset.

**Table 4:** Attack distribution of subset of the CSE-CIC-IDS2018 dataset.

Category	Training	Testing	Total
Benign	751,849	322,493	1,074,342
Attack	203,252	86,837	290,089

### Preprocessing

As discussed above, raw dataset might not yield good results due to class imbalance, various data types and huge number of input features. Therefore, to develop robust machine learning models, input features were preprocessed to overcome the challenges. In the following section, an approach for dataset preprocessing is discussed in detail. The preprocessed datasets were later used in training machine learning algorithms.

### Feature normalization

To develop a robust machine learning classifier, data normalization is required. It is the process of standardization of input data features to remove the biasness of the machine learning classifier. The data values are standardized by the maximum value in that data feature so that they lie in between standard values of (0–1). The normalization operation was applied by using Eq. (1). As a result of this process, all the numerical values were converted to a value range of 0 to 1.

(1) $${X}^{\prime} = \; \displaystyle{{x - \mu \; } \over \sigma }$$

In this equation, x is the original feature value, where X′ is the normalized value. $\sigma$ and $\mu \;$are standard deviation and mean respectively. Due to the normalization process, some features with high numerical values cannot affect the performance negatively. In addition, only 13 out of 41 features were used for final classification, in the case of NSL-KDD dataset. The detail of feature selection is provided in the next section.

### Feature selection

There are total of 41 input features in the NSL-KDD dataset, while there are 45 and 73 input features in the UNSW-NB15 and CSE-CIC-IDS2018 datasets, respectively. Selected target labels might not be affected by some input features. Keeping this in view, only those features were retained which were required for classification purposes. For this task, RFE was utilized for feature selection (Guyon et al., 2002), which is a wrapper based backward feature selection method. In this technique a model is built using an entire set of input features. Then, the important score for each feature is calculated. In this recursive process, the least important features were removed by retraining models on various sets of features. Here, a subset of feature size is a tuning parameter to calculate specific number of features. When a subset gives optimal performance, that subset is used for predictors. The final optimal subset is used to train the final algorithm. In simple words, RFE performs a greedy optimization search to find the best performing feature subset for final model. Algorithm 1 describes the feature selection based on RF-RFE method.

**Algorithm 1:** For feature selection based on RF-RFE.

**INPUT**:
Training Data D0 = [*d*_*1*_, *d*_*2*_, *d*_*3*_*…d*_*n*_]
Set of n features F = [*f*_*1*_, *f*_*2*_*,…f*_*n*_]
Subset of features S = [1,2,3,4,5….*m*]
**OUTPUT**:
Final Optimal Feature Set Fs
**BEGIN**:
S = [1,2, 3….,_ m_]
F_s_ = []
** While** S ≠ [] Do
Repeat **For** *x* in [1:*n*]
Ranking features *via* M(D,F) *#Applying RF-RFE on input data and features*
S(f*) ← F’s last ranked features
F_s_(*n* – *x* + 1) ← S(f*)
S(F_s_) ← S (F_s_) – S(f*)
** End While**
**End**

All models cannot be paired with RFE method, because when number of features increase as compared to the number of samples, some models cannot be used with RFE. RFE with RF is used in this study for two reasons. First, RF does not tend to exclude the variables from prediction equation. An ensemble method usually has increased performance as compared to the individual models. RF, being an ensemble method, enforces the trees to have sub-optimal splits of features using random sample of input features. Second reason to include RF as base learner in RFE is because it has internal mechanism to measure the importance of features (Chen et al., 2018). Other than RF-RFE feature selection method, various other methods are widely utilized by the researchers. For example, in Gulla et al. (2020) researchers applied a combination of Gray Wolf Optimization (GWO) and Particle Swarm Optimization (PSO), however the results from this study are superior as compared to the Gulla et al. (2020) and a study in Roy et al. (2022) where k-best method was utilized. This research experimented with several methods for feature selection, however in this study only results of best performing method *i.e*., RF-RFE are discussed.

**Figure 2:**
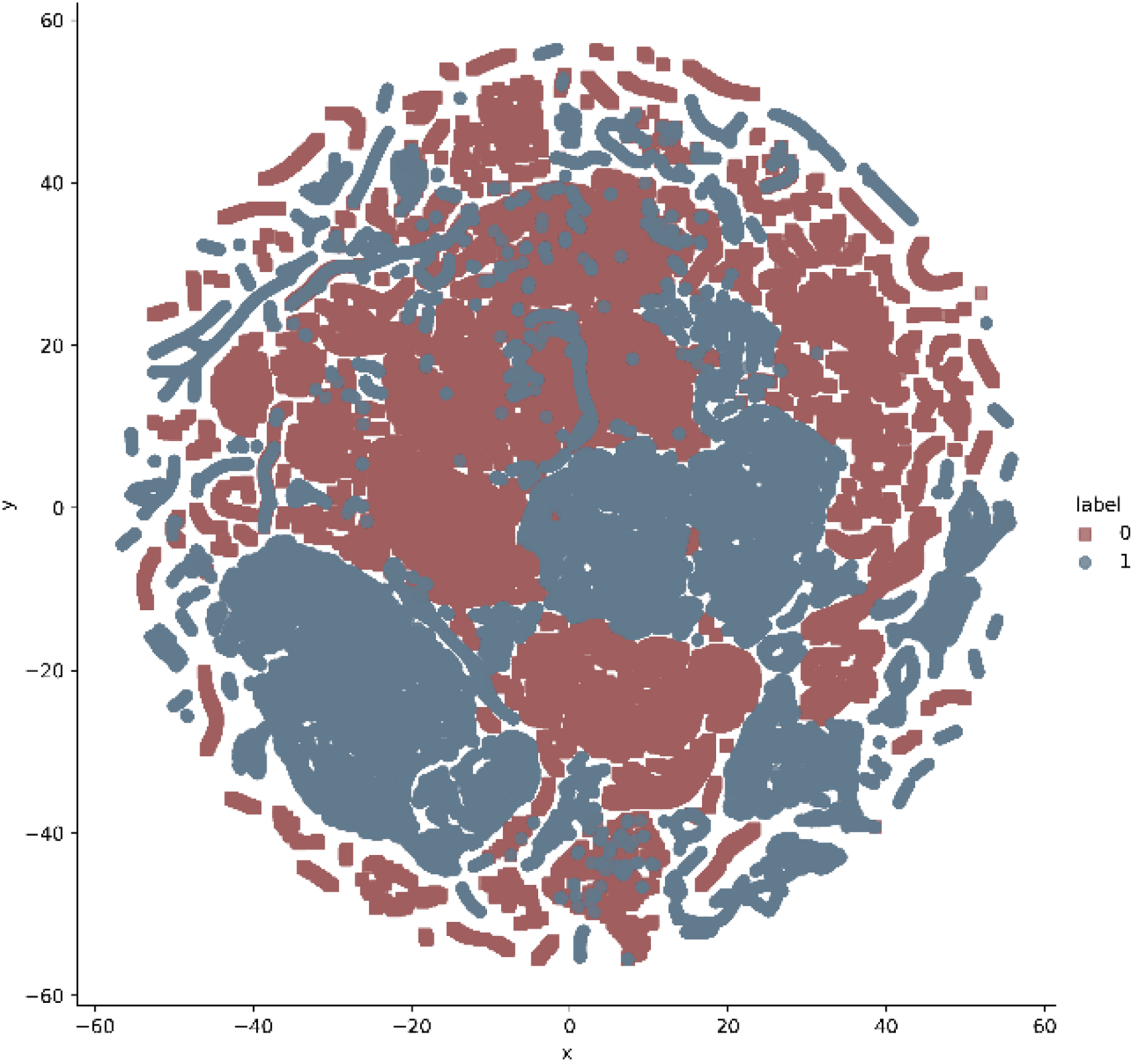
t.SNE visualization—attack *vs* normal classes on NSL KDD.

**Figure 3:**
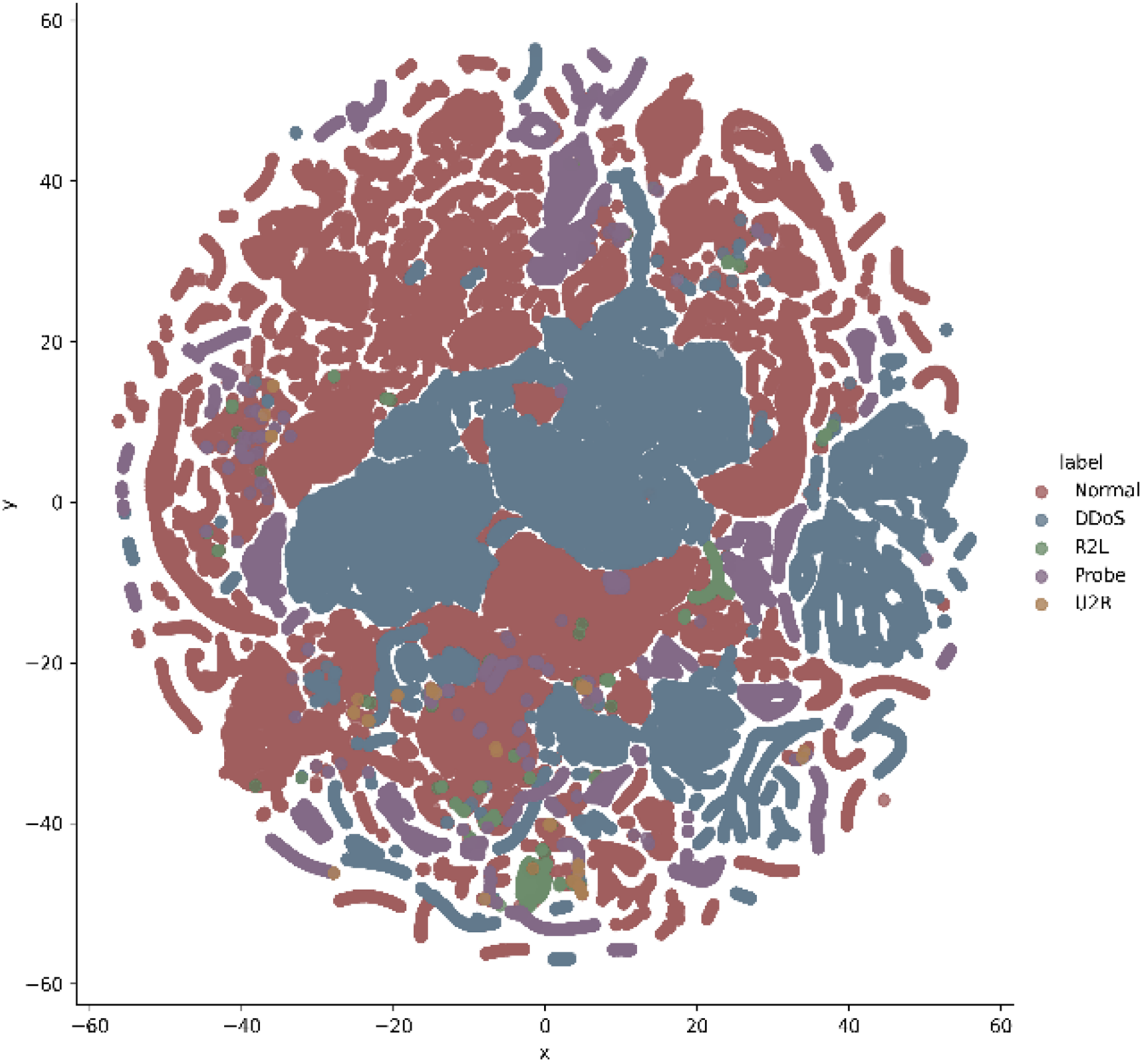
Multiclass t-SNE visualization on 41 features of NSL-KDD.

**Figure 4:**
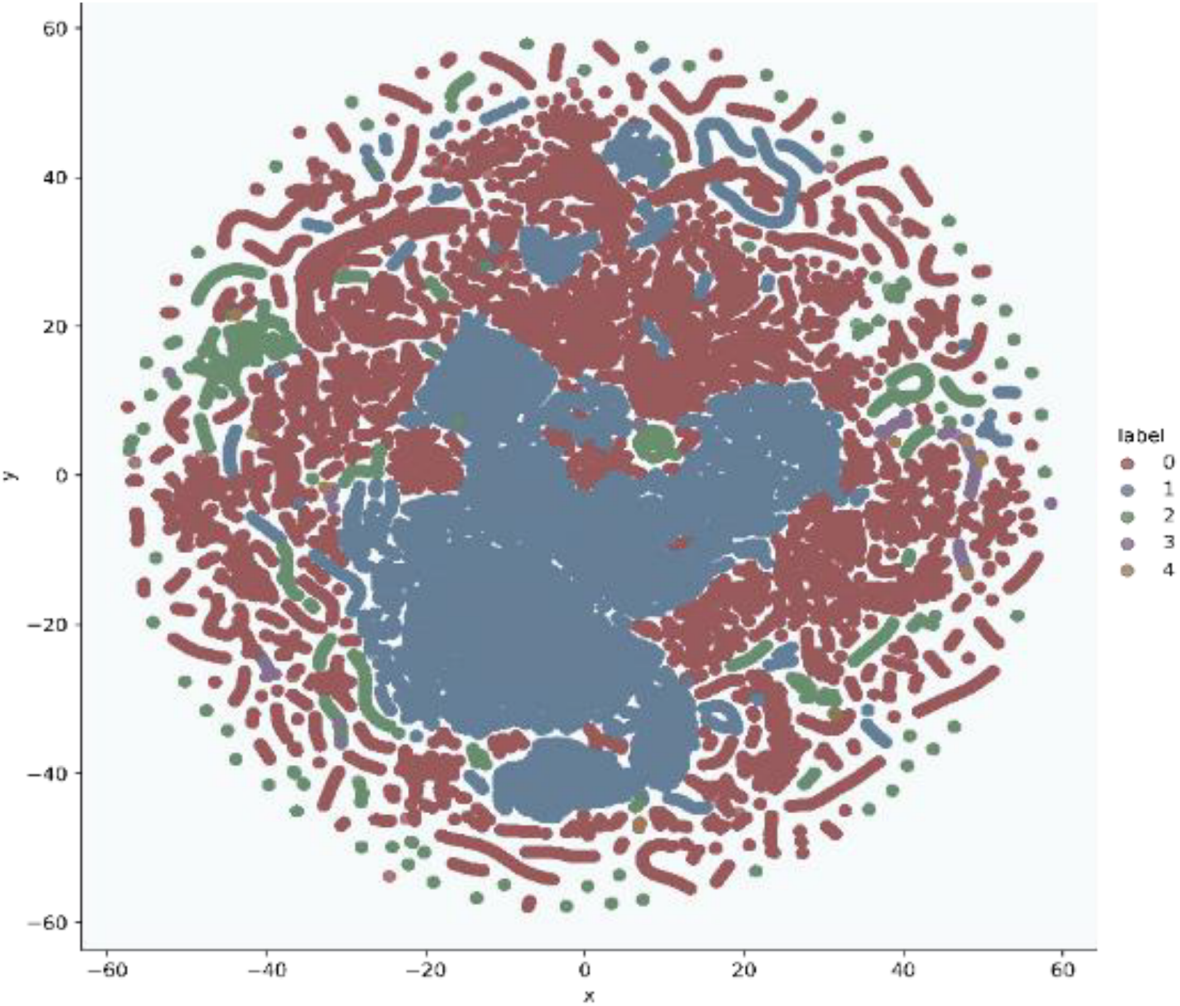
Multiclass t-SNE visualization on 13 features of NSL-KDD.

In addition, t-stochastic neighbor embedding (t-SNE) visualization was also used to visualize 41 features as shown in Figs. 2 and 3. T-distributed stochastic neighbor embedding (t-SNE) is a statistical data visualization method for high dimensional data. The method visualized the data points by giving each datapoint a location in a three-dimensional space. The method is based on stochastic neighbor embeddings. It is a nonlinear technique for data dimensionality reduction. It can be seen from the visualizations that the red dots overlap the blue and other dots representing different kinds of attacks in different regions. It becomes difficult for the learning algorithm to learn various distribution areas for the same type of attack traffic at the same time. The RF-RFE algorithm, applied to data, suggested only 13 features that were important for classification. In Fig. 4, t-SNE visualization for features selected by RF-RFE algorithm for final classification is shown. Features selected by RF-RFE help to differentiate the attack types and decreases the overlap between attacks.

### Machine learning algorithms

Most ML algorithms need extracted features while deep learning, which is a subset of machine learning, does not require extracted features as it directly learns from the data. Instead of direct implementation of popular algorithms, in some cases, algorithms are merged to form ensemble models. In this research study, five individual ML models and three deep learning models along with proposed ensemble method were trained. Further details have been discussed in the upcoming sections.

### Hyperparameters values

Hyperparameter values are necessary in machine learning approaches to train the proposed model. For deep learning models, basic and simple architectures were trained and tested to overcome overfitting as deep learning algorithms easily overfit on the training data. Architecture engineering in deep learning itself is a huge research domain. So, only training of basic variant of deep learning algorithms was considered and training the optimized deep learning algorithms for network intrusion detection was seen outside the scope of this work. Table 5 summarizes the key information about hyperparameters values used in the present research.

**Table 5:** Hyperparameters values to train the algorithms.

Classifier	Parameters
RF	Number of Trees = 10; Max Features = 13
KNN	Number of Neighbors = 5; Algorithm Solver = Auto
SVM	Kernel = Linear and Poly (for multiclass) ; Regularization parameter (C = 1.0)
DT	Max Depth = Auto; Max Features = 13
MLP	Number of iterations = 300; Hidden Layer Size = 100; Activation = ReLU; Optimizer = Adam; Learning Rate = 0.001
CNN	Epochs = 30, Optimizer = Adam, Conv1D layers = 4, Loss Function = Sparse Categorical Cross Entropy, Batch Size =128, Learning Rate = 0.001
RNN	Epochs = 30, Optimizer = Adam, RNN layers = 3, Loss Function = Sparse Categorical Cross Entropy, Batch Size =128, Learning Rate = 0.001
LSTM	Epochs = 30, Optimizer = Adam, LSTM layers = 3, Loss Function = Sparse Categorical Cross Entropy, Batch Size =128, Learning Rate = 0.001

### Ensemble method

The main idea behind the ensemble learning is to get the advantage from various classifiers by learning in a collective ensemble way. Each classifier has its own strengths and drawbacks for data classification. Some classifiers perform well on specific types of attacks while others may perform well on the rest of attack types. The key idea is to combine several weak classifiers by training multiple classification models and develop a strong classification model by utilizing a voting algorithm. In this way, weaknesses of the classification algorithms can be reduced to develop a strong classification model. The proposed ensemble model is based on RF, MLP and SVM classification models. These algorithms were trained collectively *via* a hybrid approach where RF-RFE helped to reduce the input features and later, a stacking ensemble method used the individual predictions to calculate the final class prediction using majority vote. The proposed method helped in minimizing the variance and increasing the predictive force of collective learners. The results showed that the performance gains were notable when utilizing the proposed ensemble method.

**Figure 5:**
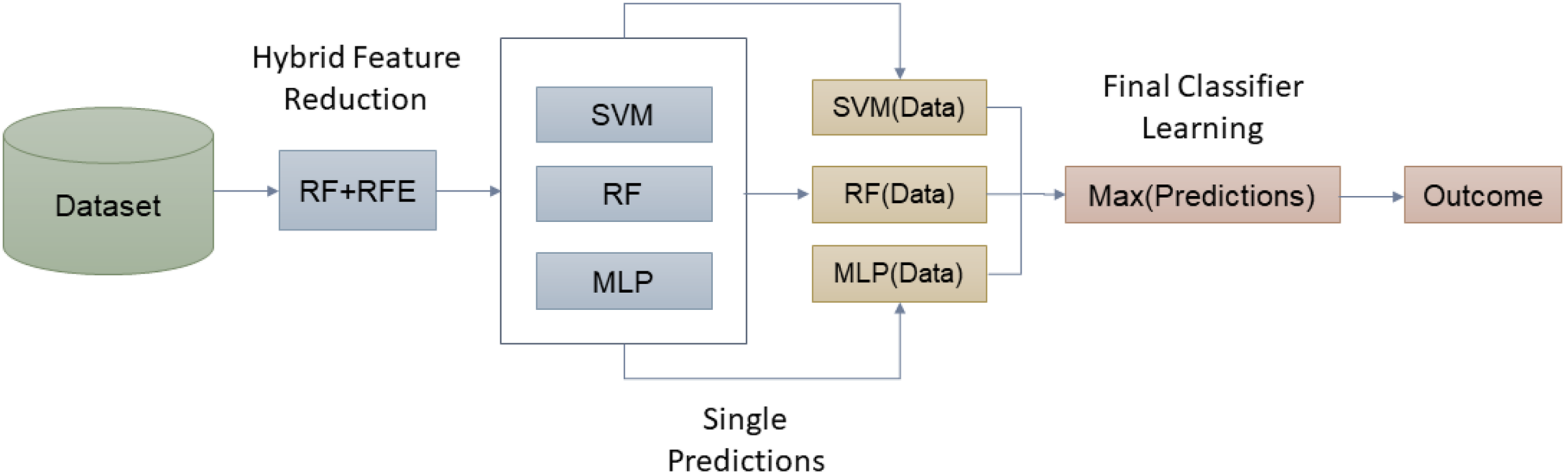
Working of proposed hybrid ensemble based on RF-RFE.

Figure 5 presents the basic idea of the proposed approach in this article. Algorithm 2 presents the steps in the proposed ensemble model training and testing.

**Algorithm 2:** Proposed ensemble classifier.

**INPUT**:
Training Data N × k (k Features selected by Algorithm 1 with N observations)
Testing Data N × k
**OUTPUT**:
Trained Ensemble Classifier E_c_
Final Attack Predictions using E_c_
**BEGIN**:
/* Training Step */
Initiate Ensemble Models
Algorithms = [MLP, SVM, RF]
Trained_Models = []
**For** i=1 to N:
** For** model in Algorithms:
Train model on k Features
Trained_Models ← model
** End For**
**End For**
/* Testing Steps*/
predictions = []
**For **model in Trained_Models
pred **=** model(Test Data)
Predictions ← pred
**End For**
final_prediction = E_c_(predictions) *#Ec selects the final class based on majority predictions*
**END**

### Evaluation metrics

Several evaluation methods have been used to evaluate the performance of classifiers. Most of the metrics come from the information retrieval domain. Some of the popular metrics to evaluate the classifier are precision, recall, accuracy, F1-score, and mean absolute error (MAE). In this section, various evaluation metrices for classifiers’ evaluation are described. Many of these metrics can be calculated by using a confusion matrix. A typical confusion matrix is presented in Table 6. This confusion matrix forms the basis of various other metrics to evaluate the final classifier. The correct and incorrect predictions can be presented in a tabular form and a confusion matrix is generated. TN, TP, FP, FN are four values which are part of a confusion matrix.

**Table 6:** Confusion matrix.

Predicted			
Actual class label		Attack	Normal
	Attack	*TP*	*FN*
	Normal	*FP*	*TN*

***Accuracy***—It is the percentage of the correct predictions by the classifier. It can be calculated from the terms that are defined earlier in the confusion matrix section. It can be calculated by using a following formula:

(2) $$Accuracy = \displaystyle{{TP + TN} \over {TP + FP + TN + FN}}$$

***Precision***—It indicates the probability of a test instance that was positive and correctly identified as positive. It is given by:

(3) $$Precision = \displaystyle{{TP} \over {TP + FP}}$$

***Recall***—It is also called true positive rate. It gives us the indication of actual positive values which were identified correctly. It can be calculated by following formula:

(4) $$Sensitivity = \displaystyle{{TP\; } \over {TP + FN}}$$

***F1-Score***—It is the harmonic mean of sensitivity and precision. The final classifier can be evaluated using this evaluation metric. It can be calculated as:

(5) $$F1\; Score = 2*\displaystyle{{Precision*Recall\; } \over {Precision + Recall}}$$

## Results and discussions

The proposed machine learning model has been developed and trained in Python programming language using Sklearn, Keras, Pandas, and NumPy libraries. Five machine learning models and three deep learning along with proposed ensemble model have been developed in this research work. These models are trained using the preprocessed NSL-KDD, UNSW-NB15 and CSE-CIC-IDS2018 datasets to select the best performing mode. All experiments were done on Intel core i5 processor on HP 840 G2 laptop with 64-bit Windows 10 operating system, and 16 GB RAM.

### Evaluation using NSL-KDD dataset

The detail of results for NSL-KDD with classification performance on original set of forty-one features is presented in Table 7. Results showed that for all features used, classifiers did not give higher percentages on the performance metrices. With all features utilized, the ensemble method showed better performance than the rest of the classifiers, yet the overall performance was not appreciable. For the next stage, classification performance based on feature selection method was investigated. In total, thirteen features were selected out of the original forty-one features in the dataset. The results showed that random forest classifier achieved an accuracy of 98.81% with 97.70% precision value. The recall and F1-score were obtained as 95.66% and 96.67% respectively. The lowest accuracy was achieved in the case of K-Nearest Neighbor algorithm with 94.72% accuracy score. This could be due to the reason that it is a very simple method and could not handle complex data. The overall details of the obtained results can be seen in the Table 8.

**Table 7:** Classification performance on original NSL-KDD dataset (41 features).

Classifier	Accuracy	Precision	Recall	F1-measure
Random forest	71.91%	68.76%	72.66%	70.65%
KNN	68.71%	67.61%	69.25%	68.42%
SVM	74.32%	72.19%	69.92%	71.03%
Decision tree	67.39%	66.21%	65.43%	65.81%
MLP	78.32%	79.43%	77.14%	78.26%
Ensemble	82.91%	81.82%	84.78%	83.27%

**Table 8:** Classification performance based on feature selection method for NSL-KDD dataset (13 features).

Classifier	Accuracy	Precision	Recall	F1-measure
Random forest	98.81%	97.70%	95.66%	96.67%
KNN	94.72%	95.61%	93.51%	94.53%
SVM	98.33%	96.11%	98.40%	97.23%
Decision tree	96.35%	95.11%	96.42%	95.74%
MLP	98.32%	97.43%	98.14%	97.78%
Ensemble	99.53%	99.79%	98.78%	99.29%

**Figure 6:**
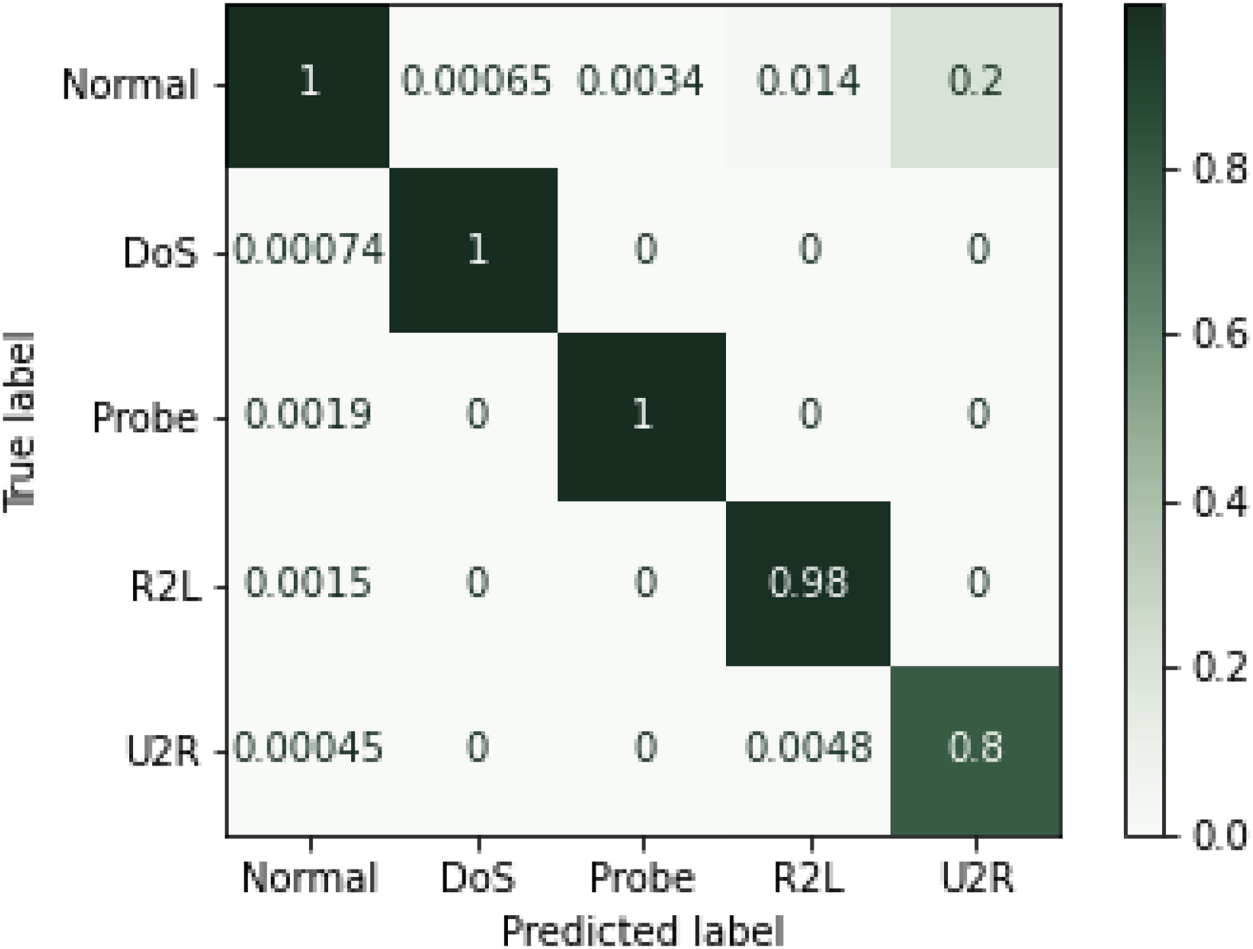
Confusion matrix for NSL-KDD (Normalized).

Experimental results showed that RF outperformed other algorithms in terms of accuracy and precision measures. The same trend can be seen in the literature where random forest performed better as compared to the other algorithms (Taher, Jisan & Rahman, 2019). There might be two reasons for these results. First, it works by voting mechanism where predictions from various decision trees are used to make final decision. The second reason could be the fact that by having multiple decision trees in the forest, only best features that contribute to the final prediction are selected for training the final classifier. Although MLP showed better results as a single classifier, when used in an ensemble model, considering its extensive training time, preference was given to KNN. Conclusively, ensemble of RF, SVM and MLP performed undoubtedly well with less training time and high percentages on performance metrices. Hence, efficient network intrusion detection systems can be developed by utilizing high performance machine learning algorithms. Confusion matrix for the NSL-KDD dataset is given in the Fig. 6 below.

**Figure 7:**
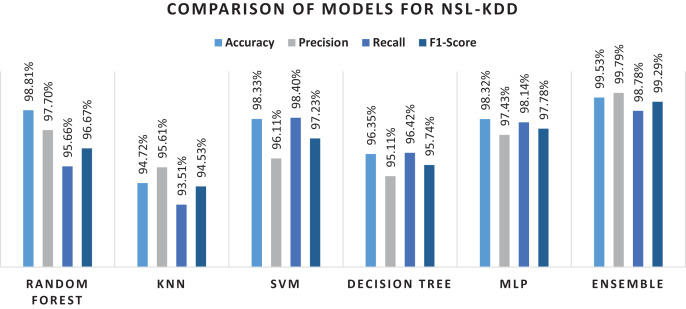
Results of selected machine learning models for network intrusion detection on preprocessed data.

Overall performance of machine learning algorithms has been shown through a bar graph in the Fig. 7, while performance of various machine learning methods on NSL-KDD dataset is shown in Table 8 shows result of each classifier with performance metrics mapped on the attacks included in NSL-KDD dataset. For each attack type, ensemble model outperformed individual algorithms. However, a slight variation was observed in recall and F1-score metrics for U2R and Probe attack. The trees in RF protect each other from their individual prediction errors. Although some trees could be wrong but many others will be correct at the same time. Therefore, the trees as a group can make the right prediction by helping each other in the prediction task. On other hand, the ensemble method is based on heterogeneous based classifiers which do not show similar behavior and the number of attacks in Probe and U2R are very limited. Due to this, RF performed better as compared to the method on U2R and Probe. Still, the proposed method was found much better as compared to the presented methods in the related literature.

Table 9 shows result of each classifier with performance metrices mapped on the attacks included in NSL-KDD dataset. For each attack type, ensemble model outperformed individual algorithms. However, a slight variation was observed in recall and F1-score metrics for U2R and Probe attack. The trees in RF protect each other from their individual prediction errors. Although some trees could be wrong, but many others will be correct at the same time. Therefore, the trees as a group can make the right prediction by helping each other in the prediction task. On other hand, the ensemble method is based on heterogeneous based classifiers which do not show similar behavior and the number of attacks in Probe and U2R are very limited. Due to this, RF performed better as compared to the proposed method on U2R and Probe. Still, the method was much better as compared to the presented methods in the related literature.

**Table 9:** Performance of various machine learning methods on NSL-KDD (Binary classification).

Attack type	Algorithm	Accuracy	Precision	Recall	F1-measure
DoS	KNN	0.997	0.996	0.996	0.996
	SVM	0.993	0.991	0.994	0.992
	Decision tree	0.994	0.991	0.994	0.992
	MLP	0.996	0.991	0.994	0.992
	Random forest	0.995	0.992	0.996	0.997
	Ensemble	0.998	0.998	0.997	0.997
Probe	KNN	0.990	0.986	0.985	0.985
	SVM	0.984	0.969	0.983	0.976
	Decision tree	0.995	0.969	0.983	0.976
	MLP	0.991	0.969	0.983	0.976
	Random forest	0.991	0.996	0.992	0.995
	Ensemble	0.997	0.987	0.989	0.988
R2L	KNN	0.967	0.953	0.954	0.953
	SVM	0.967	0.948	0.962	0.951
	Decision tree	0.979	0.948	0.962	0.949
	MLP	0.973	0.948	0.962	0.955
	Random forest	0.992	0.964	0.837	0.903
	Ensemble	0.973	0.959	0.964	0.961
U2R	KNN	0.997	0.931	0.850	0.878
	SVM	0.996	0.910	0.829	0.848
	Decision tree	0.996	0.910	0.829	0.848
	MLP	0.997	0.910	0.829	0.848
	Random forest	0.971	0.962	0.971	0.970
	Ensemble	0.9972	0.943	0.872	0.895

**Figure 8:**
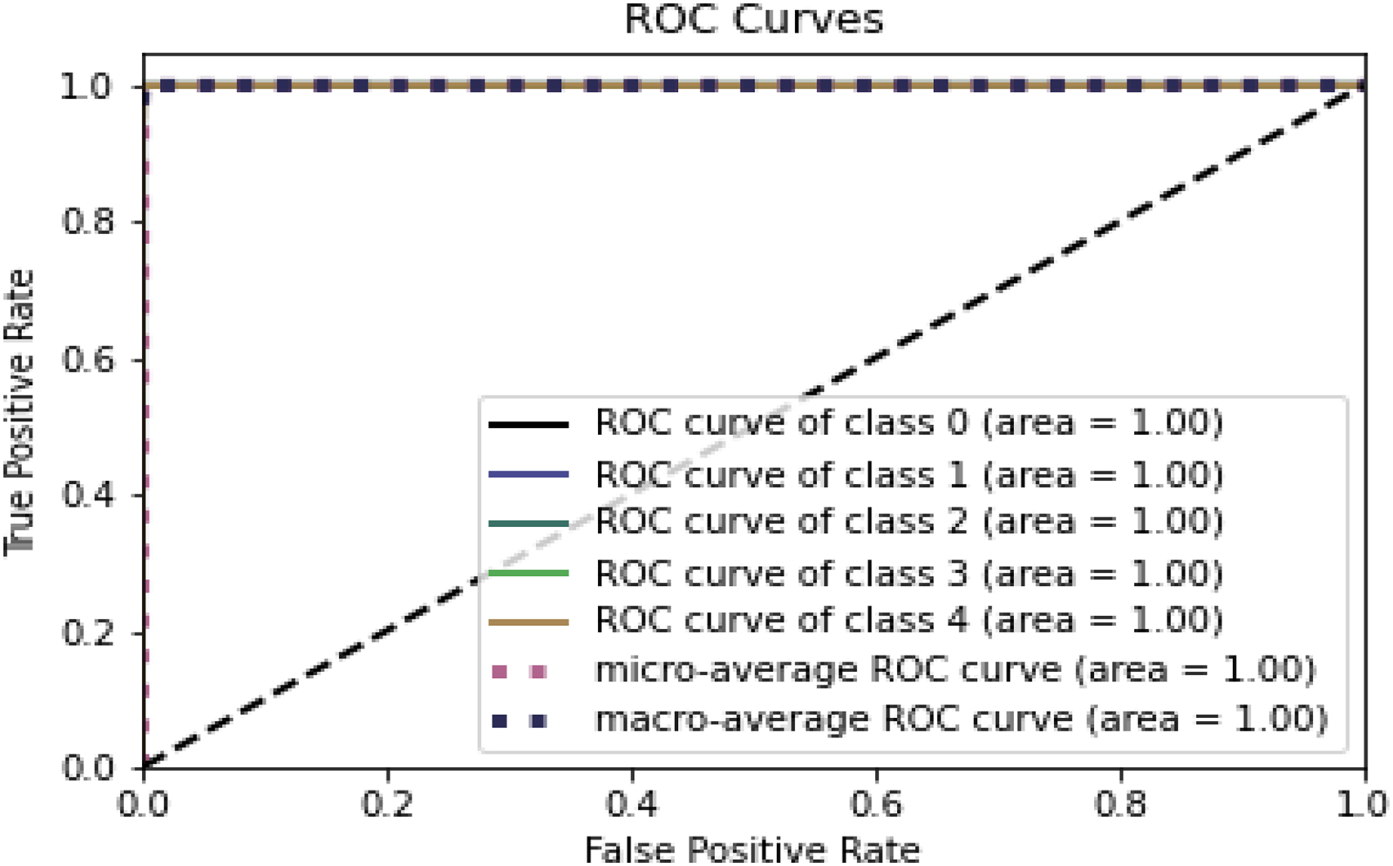
ROC for training dataset on NSL-KDD.

**Figure 9:**
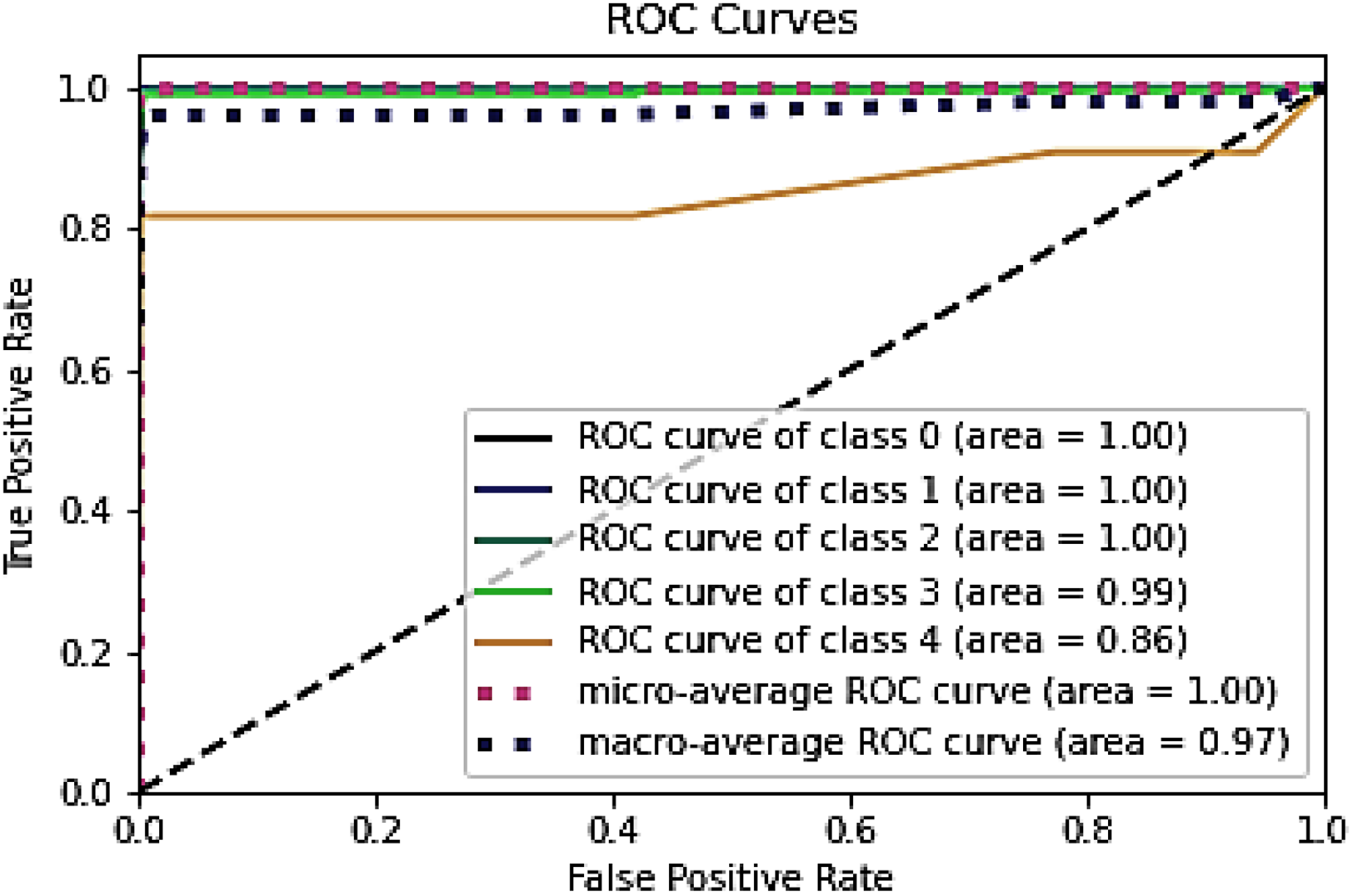
ROC for testing dataset on the NSL-KDD.

Figures 8 and 9 represent the ROC curve for the proposed ensemble model on the NSL-KDD dataset. It shows that the TPR rate is closer to 1, which is high, while the FPR is low as desired.

Table 9, Figs. 8 and 9 conclude that an IDS, using the proposed model, can effectively detect network intrusions.

Table 10 presents performance of the proposed system with some recent research works that utilized deep learning-based models for building intrusion detection systems using the NSL-KDD dataset. For a fair comparison, experiments with deep learning algorithms were carried out to demonstrate the superiority of the approach. It can be seen that the proposed approach consistently outperformed the deep learning algorithms in terms of performance measures, training time and testing time. One interesting observation was the lower model size of RNN trained model as compared to the proposed ML ensemble trained model in this study’s experiments.

**Table 10:** Comparison of recent approaches for intrusion detection on the NSL-KDD dataset (training time on whole dataset while testing time on single data sample).

Study	Method		Performance measures (%)				Multi-class	No of features	Time required (s)		Model size
	Feature selection	Classifier	Accuracy	Precision	Recall	F1-score			Training time	Testing time	
Proposed	RF-RFE	ML ensemble	99.53	99.79	99.78	99.29	✓	13	~18	0.003	~242 kb
		CNN	95.04	95.13	95.11	95.02	✓		~390	0.16	~1,024 kb
		RNN	89.3	88.10	89.12	88.19	✓		~360	0.085	~225 kb
		LSTM	91.21	91.1	91.2	91.23	✓		~800	0.084	~1,240 kb
Otair et al. (2022)	GWO+PSO	KNN + SVM	98.97	–	–	–	X	20	~1,680	0.15	–
Roy et al. (2022)	–	RF	98.5	–	–	–	✓	–	~454	~0.0030	–
Gu & Lu (2021)	k-Best	RF + XGB + DT	99.9	99.8	99.9	99.9	X	20	~8.21	0.0055	–
Pokharel, Pokhrel & Sigdel (2020)	CNN	AE	85.51	97.62	68.90	–	X	–	~1,800	0.054	–

### Evaluation using the UNSW-NB15 dataset

To further evaluate the performance of the proposed approach, the preprocessed dataset was used. Tables 11 and 12 presents the experimental results of the proposed approach on this dataset.

**Table 11:** Classification performance on the original UNSW-NB15 dataset (45 features).

Classifier	Accuracy	Precision	Recall	F1-measure
Random forest	87.91%	86.76%	85.66%	86.20%
KNN	65.71%	66.61%	67.25%	66.92%
SVM	73.32%	71.19%	69.12%	70.13%
Decision tree	78.39%	76.21%	75.43%	75.81%
MLP	77.32%	78.43%	77.14%	77.77%
Ensemble	89.91%	86.82%	85.78%	86.29%

**Table 12:** Classification performance on the UNSW-NB15 dataset with RF-RFE features (15 features).

Classifier	Accuracy	Precision	Recall	F1-measure
Random forest	97.73%	98.01%	98.02%	98.01%
KNN	86.50%	82.31%	87.41%	84.78%
SVM	84.60%	78.11%	85.40%	81.59%
Decision tree	97.58%	98.24%	98.12%	98.17%
MLP	74.21%	77.43%	77.14%	77.78%
Ensemble	98.53%	98.79%	98.78%	98.78%

**Figure 10:**
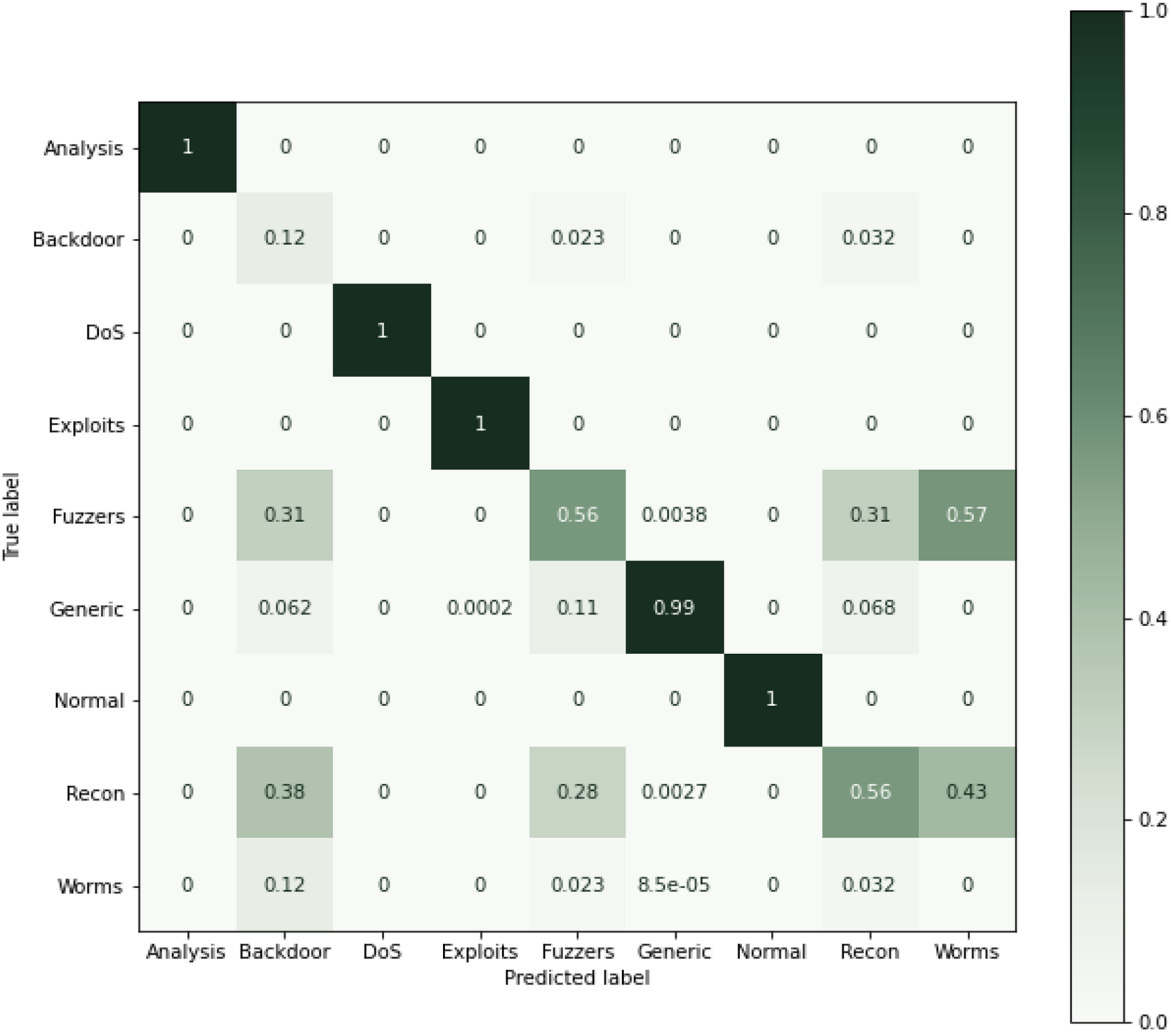
Confusion matric for the UNSW-NB15 dataset (Normalized).

As can be seen from Table 9, using all the features for classification, results in performance degradation. This is intuitive as some features are not always important for the classification. On the other hand, the results of this study suggested that ensemble performs well and shows the superior results, as seen in Table 9. The results show that RF performs better when comparing the individual algorithms with 87.9% accuracy. The lowest accuracy score was obtained in the case of KNN that was also the least performing algorithm in case of NSL-KDD. This reason for poor performance is discussed in the previous section. Reason for RF better performance is also already discussed in the previous section. Therefore, RF was chosen in the hybrid stacking ensemble method Table 12 shows the superiority of the proposed approach as compared to individual machine learning algorithms when using the proposed RF-RFE and the stacking-based ensemble method. The proposed method yields more than 98% accuracy which outperforms RF and other algorithms. Figure 10 shows the confusion matrix obtained for the whole dataset.

**Figure 11:**
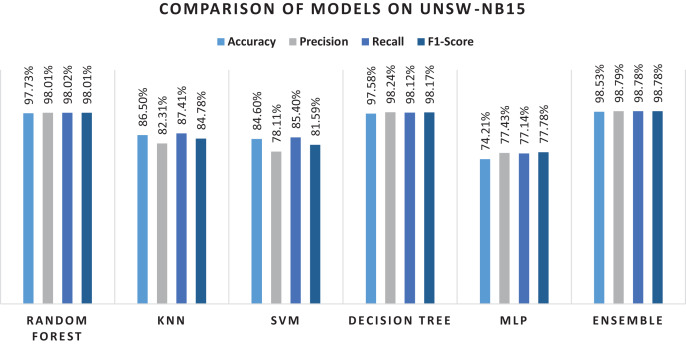
Results of selected ML models for network intrusion detection on the UNSW-NB15 dataset.

Overall performance of the algorithms and the ensemble approach is shown in the Fig. 11. The figure explains that the proposed ensemble method is outperforming all the individual algorithms in terms of classification metrics.

**Figure 12:**
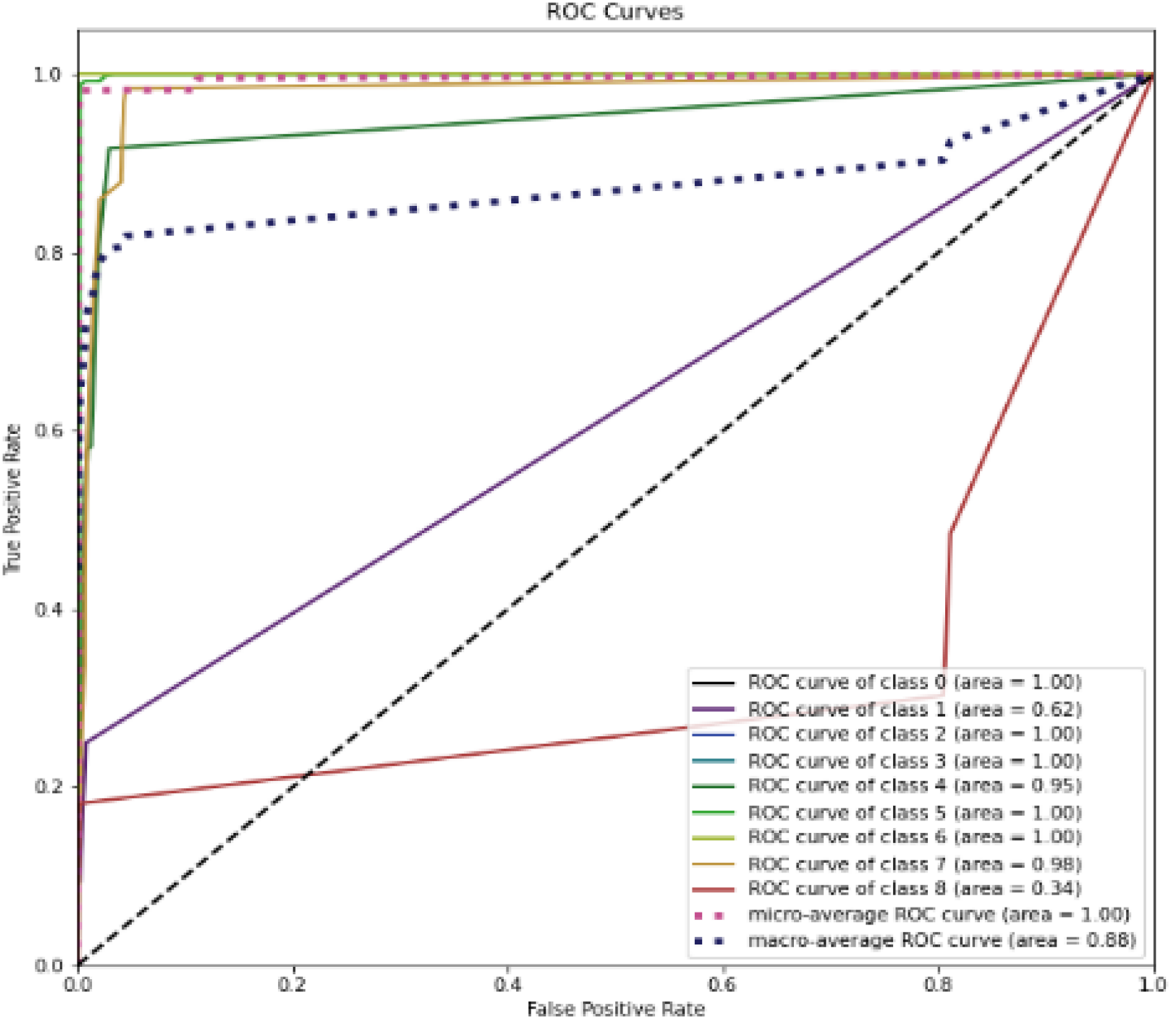
ROC curve for the UNSW-NB15 dataset.

Figure 12 represent the ROC curve for the proposed ensemble model on UNSW-NB15 dataset. It shows that the TPR rate is closer to 1 which is high for majority classes, while the FPR is low as desired. Therefore, from the results presented in Table 12 and ROC figure, it may be concluded that an IDS, using the proposed approach, can effectively detect network intrusions. Figure 12 also show that ROC area under curve is nearly 1 for most classes which shows that the proposed method can effectively differentiate between these classes. However, in the case of class 1 (Backdoor) and class 8 (Worms) a sharp decline can be seen in the area under curve. This could be due to two reasons. Firstly, the dataset is highly imbalanced, and it has few samples as compared to other majority classes. Secondly, the dimensions in the overall dataset have been reduced using RF-RFE. This might have caused loss of important features that can increase performance for these minority classes. However, overall, the proposed method performs well as compared to the individual algorithms.

Table 13 compares performance of the proposed system with some recent research works that utilized the UNSW-NB15 dataset for intrusion detection. The researchers additionally compared the results with the three deep learning algorithms as well. Researchers have used deep learning, and machine learning-based models for building intrusion detection systems. This proposed method clearly outperforms previous works by considerable margins. Other than the model size of the RNN trained algorithm, this approach outperformed deep learning algorithms that were trained on the same machine. Mostly researchers only focused on binary classification therefore results in the study (Injadat et al., 2020) have better results as compared to the results of this study. However, information about attack classes is important in determining the countermeasures in case of intrusion. Therefore, superior results in binary classification might not be helpful in determining the intrusion countermeasures. In addition, mostly researchers do not provide the features that they used for classification and training and testing time requirements. As SMEs have limited resources, therefore, training and testing time might help them in selecting better choices for IDS development with limited resources and expertise.

**Table 13:** Comparison of recent intrusion detection approaches on the UNSW-NB15 dataset (training time on whole dataset while testing time on single data sample).

Study	Method		Performance measures (%)				Multi-class	No of features	Time required (s)		Model size
	Feature selection	Classifier	Accuracy	Precision	Recall	F1-score			Training time	Testing time	
Proposed	RF-RFE	ML ensemble	98.53	98.79	98.78	98.78	✓	15	~104	0.006	~544 kb
		CNN	98.01	97.01	98.13	98.12	✓		~413	0.08	~1,029 kb
		RNN	98	97	98	97	✓		~228	0.07	~250 kb
		LSTM	98	98	98	98	✓		~390	0.071	~978 kb
Belouch, Hadaj & Idhammad (2018)	CNN	CNN + Dynamic autoencoder	98.5	98.4	98.6	98.5	X	–	–	–	~923 kb
Taher, Jisan & Rahman (2019)	IG, PSO, GA	KNN + RF	99.96	99	99	99	X	–	–	–	–
Roy et al. (2022)	–	SVM	93.75	–	–	–	X	–	~500	–	-
Dora & Lakshmi (2022)	K Best	RF + XGBoost + DT	93.7	94.5	90.2	92.29	X	20	~11.65	8 (µs)	–

### Evaluation using CSE-CIC-IDS2018 dataset

As stated in “Dataset Description”, this dataset was only considered for binary classification (benign *vs* malicious) as the data is imbalanced with fewer records for specific classes. Additionally, the dataset is divided into a few files where each file is a record with the name of the day the record was generated. Combining the data into one file requires huge computational power and processing such a massive dataset was not possible due to resource limitation. Therefore, only binary classification is considered. Further details of the dataset are already discussed in “Dataset Description”.

Table 14 provides classification performance of the algorithms using original dataset features. In the case of this dataset, RF again performs better and KNN and MLP does not perform well. In comparison to individual algorithms, the ensemble method performs better. However, performance is still low as compared to the ensemble approach using RF-RFE selected features. In the case of original features RF performance is comparable to the proposed ensemble method in terms of precision and recall. This is also confirmed from the literature (de Souza, Westphall & Machado, 2022) where RF performs well. In Table 15 the performance of algorithms using features selected by RF-RFE is compared. It can be seen in the table that the acquired results are again 1% to 2% superior.

**Table 14:** Classification performance based on the original CSE-CIC-IDS2018 dataset (Binary classification).

Classifier	Accuracy	Precision	Recall	F1-measure
Random forest	87.91%	88.76%	87.66%	88.20%
KNN	78.71%	76.61%	78.25%	77.42%
SVM	79.32%	78.19%	79.92%	79.04%
Decision tree	82.39%	81.21%	83.43%	82.30%
MLP	78.32%	79.43%	77.14%	78.26%
Ensemble	89.91%	89.82%	88.78%	89.29%

**Table 15:** Classification performance based on the feature selection method on CSE-CIC-IDS2018 (15 features).

Classifier	Accuracy	Precision	Recall	F1-measure
Random forest	98.91%	97.81%	96.69%	97.24%
KNN	95.72%	96.63%	94.15%	95. 73%
SVM	97.53%	95.15%	96.14%	95.64%
Decision tree	97.53%	96.21%	95.24%	95.72%
MLP	97.23%	96.34%	97.41%	96. 87%
Ensemble	99.9%	99.9%	99.89%	99.89%

**Figure 13:**
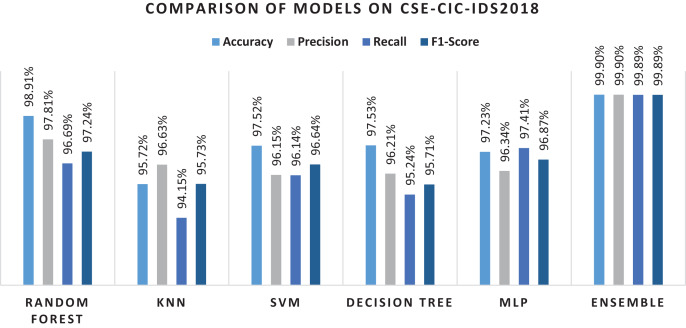
Results of selected ML models for network intrusion detection on CSE-CIC-IDS2018 data.

Figure 13 present the overall results of the machine learning algorithms and the ensemble method on RF-RFE features. Using RF as base learner in RFE helped in selecting useful features that help in final classification with few dimensions.

**Figure 14:**
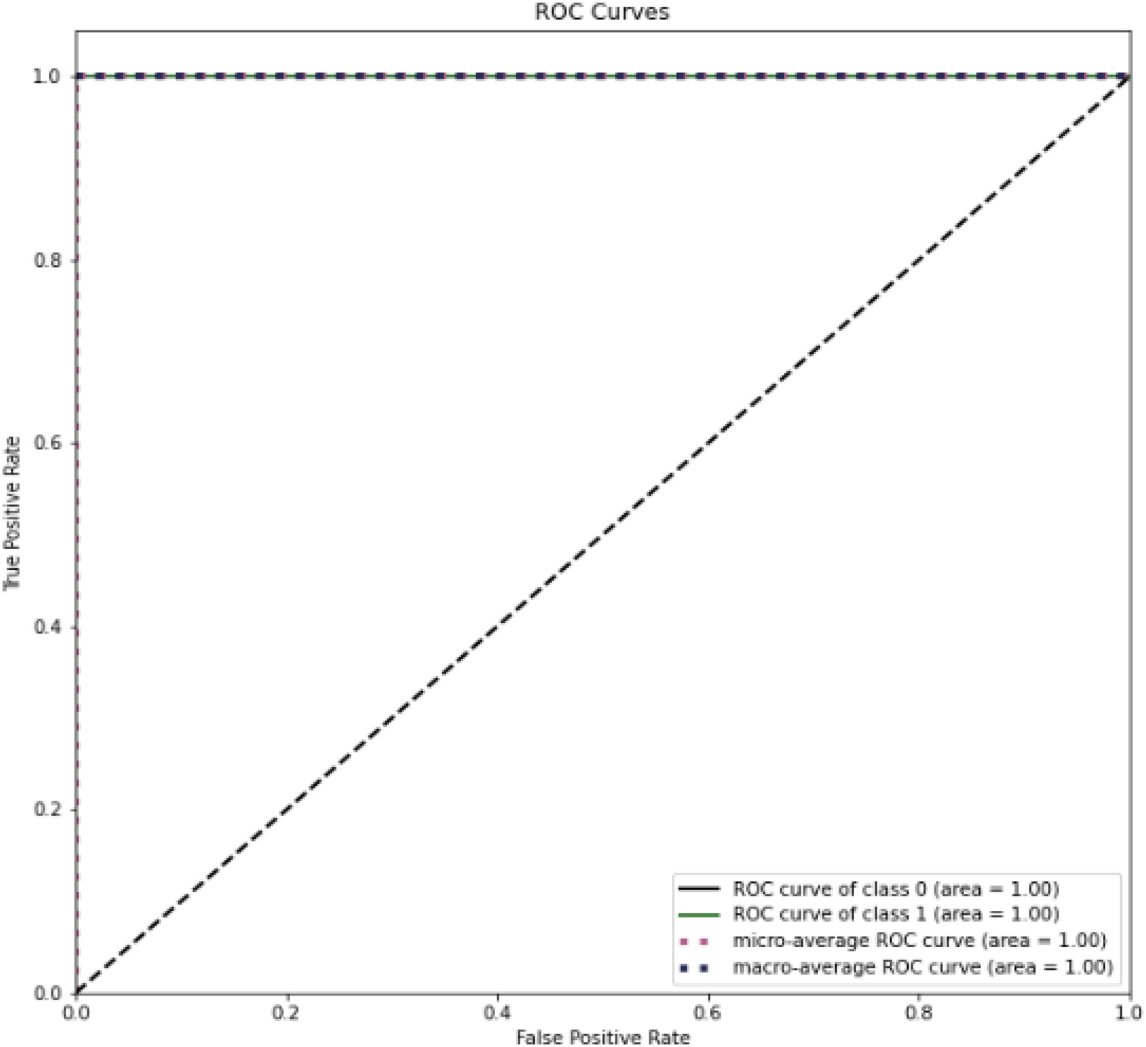
ROC curve for dataset on CSE-CIC-IDS2018.

Figure 14 present the ROC curve for testing dataset. This shows a perfect area under curve of 1 in both cases. It implies that the proposed method was able to successfully differentiate between benign and malicious attacks on network.

To demonstrate the superiority of this approach, results were compared with the recent studies that utilized a similar dataset. As stated earlier, fewer researchers have investigated CSE-CIC-IDS2018 in the experiments, whereas NSL-KDD and KDD99 have been widely used. The approach was compared with a few recently published research articles and individual deep learning models. As seen in Table 16, the proposed method has performed much better than the deep learning approaches (Kim et al., 2020). Researchers have investigated newer methods for feature selection (Abdel-Basset et al., 2021) which performs better. However, it increases the computational overhead for training and testing of machine learning systems and SMEs cannot afford such expensive methods. When compared with other studies, the proposed approach is computationally friendly, and the results are also superior. One interesting thing found is the lower size of the deep learning trained models for CSE-CIC-IDS2018 when compared to the proposed model size. However, the performance of the system is slightly better than the individual algorithms with reduced training and testing time that is a requirement for real-time systems.

**Table 16:** Comparison of recent intrusion detection approaches on the CSE-CIC-IDS2018 dataset (training time on whole dataset and testing time on single data sample).

Study	Method		Performance measures (%)				No of features	Time required (s)		Model size
	Feature selection	Classifier	Accuracy	Precision	Recall	F1-score		Training time	Testing time	
Proposed	RF-RFE	ML ensemble	99.9	99.9	99.89	99.89	15	~40.76	0.007	~912 kb
		CNN	99.2	99.2	99.1	99.1		~1,337	0.091	~220 kb
		RNN	99.9	99.7	99.8	99.7		~1,450	0.095	~240 kb
		LSTM	99.9	99.8	99.7	99.8		~2,050	0.10	~444 kb
Abdel-Basset et al. (2021)	Traffic Attention	ResNet	98.71	94.91	94.3	94.92	20	~213	1.2	–
de Souza, Westphall & Machado (2022)	Extra Tree	RF + DNN + ET	98.21	97.90	–	–	–	–	7.19	–
Kim et al. (2020)	CNN	Fully connected network	91.5	70.87	83.5	76.66	–	–	–	–

The proposed ensemble algorithm, based on hybrid feature selection, has outperformed individual algorithms on all three publicly available datasets. In case of NSL-KDD dataset, the method yielded more than 99% accuracy and F1-score that outperformed individual best performing model *i.e*., RF, while only using less than half of the original dataset features. At the same time, in comparison with the literature, the proposed method also outperformed the recent studies, as presented in Table 10. During investigation on UNSW-NB15 and CSE-CIC-IDS2018 datasets, same trends were observed where the proposed method outperformed the individual algorithms. The method yielded more than 98% F1-score and accuracy on UNSW-NB15 and more than 99% accuracy and F1-score on CSE-CIC-IDS2018 datasets. The detailed comparisons with recent studies are presented in Tables 14 and 16. The feature selection method helped in feature reduction while only selecting the best features that contributed towards the final prediction. This also helped in data dimensionality reduction and enhanced training and testing efficiency while generating state of the art intrusion detection results.

### Implications

The proposed ensemble method excelled in performance metrices as compared to the individual machine learning algorithms. Moreover, in contrast to the deep learning approaches, that require large, labeled datasets, this simple ensemble method achieved the same performance without requiring high-end GPUs. This can be particularly helpful in the edge computing and internet of things (IoT) domain. As the edge devices do not have enough computation power, therefore methods based on deep learning may not work well in real-time in case of any intrusion. The first step to prevent attacks in networks is the timely detection of that attack. As discussed, in a resource constrained environment, deep learning methods might not work in real-time, so untimely detection of network intrusions can be disruptive in such scenarios. Moreover, computationally expensive deep learning models based NID systems are not a critical requirement in small and medium enterprises. SMEs can readily implement machine learning based systems and fulfil their need of combating cybercrimes.

### Limitations and future work

To develop a machine learning based IDS, a comprehensive and representative of real-world attacks dataset is required. In this article, NSL-KDD is primarily used to perform extensive experiments. Although the results are superior on the dataset, a main challenge is to deploy the trained models on resource constrained devices such as edge devices and monitor the performance. In future, we would like to deploy and investigate the performance of such methods on edge devices.

Additionally, the UNW-NB15 dataset has been used to evaluate the performance of the proposed method. The dataset is imbalanced, and some classes have only a few handed samples (Backdoor and Worm). These minority classes affect the performance of the overall system that can be seen in the results and experimentation section. Therefore, CSE-CIC-IDS2018 dataset was further investigated to test the proposed approach. The dataset is comparatively new and contains millions of records. One problem with this dataset is the requirement for computational resources. Still, the method works well with this dataset and performs binary classification with more than 99% accuracy. Due to unavailability of computational resources, only binary classification was investigated on a subpart of the dataset. In future we would like to extend this work to the whole dataset in multiclass classification scenario.

In future, these datasets should be extended for more efficient development of network IDS. Moreover, different sectors should develop and share real-time network intrusion datasets to help researchers design sector specific intrusion detection systems. In the current study, machine learning has shown the ability to detect attacks in network. Therefore, we believe that with the development of newer intrusion datasets, a similar approach can be extended to work with the newer datasets.

## Conclusions

Defensive security has evidently become a top priority of any organizational network to safeguard against financial, reputational, and legislative exposures. Once intruded, networks and systems can be used for exploitation of vulnerabilities and transformation of a risk into an attack. This study investigates the performance of five powerful algorithms for network intrusion detection along with a newly proposed hybrid stacking algorithm-based RF-RFE feature selection method. The results showed that the proposed ensemble classifier performed equally well as compared to the other deep learning algorithms with an accuracy of more than 99.5%, 98.5% and 99.9% on the NSL-KDD, UNSW-NB15 and CSE-CIC-IDS2018 datasets, respectively. Additionally, individual selected algorithms also performed well on the benchmark datasets. The ensemble model optimizes the prediction of domain specific features and properties and contributes to designing secure networks, systems, and applications. Likewise, design solutions for secure systems and networks for varying domains can be formulated. In future, a similar approach can be extended to work with newer intrusion detection datasets.

## Additional Information and Declarations

### Data Availability

The following information was supplied regarding data availability: The code is available at GitHub and Zenodo: - https://github.com/qaixerabbas/network_intrusion_detection. - Qaiser Abbas, Dr. Sadaf Hina, Hamza Sajjad, Khurram Shabih Zaidi, & Rehan Akbar. (2023). Network Intrusion Detection. Zenodo. https://doi.org/10.5281/zenodo.7801597. The data used in this research is available at: - NSL-KDD: https://www.unb.ca/cic/datasets/nsl.html. - UNSW-NB15: https://research.unsw.edu.au/projects/unsw-nb15-dataset. - CICIDS2017: https://www.unb.ca/cic/datasets/ids-2017.html.
